# Entwined life events: The effect of parental incarceration timing on children’s academic achievement

**DOI:** 10.1016/j.alcr.2022.100516

**Published:** 2022-11-01

**Authors:** Matthew P. Fox, Ravaris L. Moore, Xi Song

**Affiliations:** a Department of Criminal Law, Max Planck Institute for the Study of Crime, Security and Law, Germany; b Department of Sociology, New York University, Princeton University, Loyola Marymount University, USA; c Department of Sociology, University of Pennsylvania, USA

**Keywords:** Child development, Crime, Parental incarceration, Academic achievement, Causal inference

## Abstract

Parental incarceration has negative effects on children’s educational outcomes. Past studies have only analyzed, and therefore only treated as consequential, parental incarceration that occurs during childhood rather than prenatally. Such analyses that emphasize the importance only of events that occur during one’s lifetime are common in life course studies. This paper introduces an “entwined life events” perspective, which argues that certain events are so consequential to multiple persons’ lives that they should be analyzed as events within multiple independent life courses; parental incarceration, whenever it occurs, is entwined across and shapes both parents’ and children’s lives. Drawing on data from the Panel Study of Income Dynamics and the Fragile Families and Child Wellbeing Study, we find that parental incarceration, both prenatal and during childhood, significantly influences children’s academic ability measures and years of completed schooling. Our results show heterogeneous effects by children’s race. We find that the absolute magnitude of parental incarceration effect estimates is largest for White children relative to estimates for Black and Hispanic children. At the same time, outcome levels tend to be poorer for Black and Hispanic children with parental incarceration experience. We explain this racial heterogeneity as confounded by the many other social disadvantages that non-White children encounter, resulting in the individual effect of parental incarceration not being extremely disruptive to their academic growth.

## 1. Introduction

Social actors live interdependent lives such that the intersection between social worlds over the life course provides a mechanism through which unique life events like birth, marriage, childbearing, and death, affect the life outcomes of others (Conger and Elder, 1994; Elder, 1994). The relative timing of these important life events has implications for both the actor and those to whom the actor is closely tied (Bengtson et al., 2012; Hogan, 1978). While these events in-and-of-themselves are life-altering regardless of when they occur, scholars often implicitly map such events to an approximate age as a proxy for biological maturation, psychological development, and membership in larger social categories (Settersten and Mayer, 1997). Because societies conceptualize age in ways that tie important experiences, roles, and statuses to specific age points (Kertzer, 1989), this age structuring is treated as an “objective biography” (Bertaux and Kohli, 1984). While a life course perspective conceptualizes our lives as embedded in both social institutions and history, it emphasizes age-graded patterns (Elder et al., 2003; O’Rand, 1996). This paper offers an alternative to both “objective biography” and the life course perspective that instead emphasizes stage-graded life events and their consequences. Using the example of incarceration timing as a criminal sanction in the United States, we examine this event’s influence on educational outcomes in another’s life course, specifically the academic achievement and long-term educational attainment of inmates’ children.

We argue for the importance of considering the relative timing of significant events in multiple life courses. Relative event timing is especially important when studying the interactions between individuals’ social worlds, as in the case of the effects of parent’s life events on children. The linked lives framework, which “… refers to the interaction between individual’s social worlds over the life span” (Elder, 1994, p. 6), has historically been the dominant frame for conceptualizing such interactions. This framework is limited by an assumption of overlapping life spans around a period or event of interest. We introduce the concept of “entwined life events” as a complementary perspective that addresses the importance of relative event timing in a secondary life course (such as a parent) on outcomes in a primary life course (such as a child).

We demonstrate this perspective by reconsidering the effects of parental incarceration on children’s academic achievement through an entwined life events framework. Within the incarceration literature, how the relative timing of parental incarceration affects child outcomes has become an important topic of discussion. Though certain studies have argued any relationship to be spurious and instead attributed significant findings to unobserved heterogeneity (Porter and King, 2015), the majority have provided evidence of parental incarceration’s impact on both short-term simultaneous and long-term subsequent child outcomes (McCauley, 2020; Niño and Cai, 2020; Turney, 2022; Turney and Lanuza, 2017), as well as that the effects on long-term outcomes are dependent upon the developmental stage that it is first experienced (Young et al., 2020). However, while these studies have begun to focus on outcomes that may fall outside “linked lives” overlapping lifespans, none have addressed the effects of parental incarceration that occurs before a child’s birth.^1^ Such a conceptualization that solely focuses on studying the effects of parental incarceration occurring during one’s lifetime ultimately treats the event as something that only affects children through a shared event experience. The entwined life events perspective goes further to admit indirect effects on kids through post-incarceration changes in the parent’s life course trajectory. This can yield effect heterogeneity on children’s outcomes conditional upon whether the parental incarceration spell preceded the focal child’s birth or occurred during childhood or beyond. Through this conceptualization, the parental incarceration event is entwined with both the parent’s and child’s life course.

Parental incarceration is a common experience in contemporary American society as over half of prisoners have children aged 17 or younger and millions of additional children have previously had a parent incarcerated (Glaze & Maruschak, 2008; Maruschak, Bronson, & Alper, 2016; Trusts, 2010). A plethora of research has established that incarceration has collateral consequences on inmates’ families, who are forced to “do time on the outside” (Braman, 2007). In comparing the children of the incarcerated with their peers, the event is inextricably linked to disparities in social capital (Clear, 2007; Wakefield and Wildeman, 2013), rates of delinquency (Roettger and Dennison, 2018), residential stability (Geller et al., 2009), earnings (Mears and Siennick, 2016) and education (Hagan and Foster, 2012a; Turney and Haskins, 2014), among other life outcomes. Yet, despite unambiguous observations of these disparities, researchers continue to debate the mechanisms through which they occur (for example, (Haskins et al., 2018; Johnson and Easterling, 2012; Wildeman et al., 2013). Education is often presented as a key driver of social mobility (Blau and Duncan, 1967; Featherman and Hauser, 1978); for reviews, see (Breen and Jonsson, 2005; Goldthorpe, 2014). Scholars have sought to unpack how and why parental incarceration affects children’s intellectual growth and educational attainment (Foster & Hagan, 2015a,b; Hagan a& Foster, 2012a). These studies show that parental incarceration affects all stages of children’s educational trajectory, including early and middle childhood grade-retention or being held over (Cho, 2009; Turney and Haskins, 2014), the likelihood of special education placement (Haskins, 2014), high school dropout rate (Trice & Brewster, 2004), eventual college completion (Hagan and Foster, 2012a) and even the educational attainment of classmates (Hagan & Foster, 2012b). Explanations proposed for these disparities have been diverse, ranging from children’s exposure to or learning from parents’ antisocial and anti-education values or norms (Williams & Godfrey, 2015; Wyse et al., 2014) to claims that teachers’ perceptions of children’s academic performance are influenced by knowledge of the incarcerated parent (Dallaire et al., 2010; Turney & Haskins, 2014).

The ability to examine the effects of parental incarceration (including on education) is hampered by the dearth of data connecting incarceration to later life outcomes, much less to the life courses of the children of the incarcerated. Admittedly, there are a number of longitudinal, nationally representative data sets that have been analyzed for these purposes, but such data relates to countries like Sweden (Hjalmarsson & Lindquist, 2012) and Denmark (Andersen, 2018; Wildeman & Andersen, 2017) where criminal incarceration is less prevalent and both the national population and persons incarcerated are more demographically homogeneous than the United States (Brodeur, 2007; Raphael, 2009; Tonry, 1999). To study the social effects of American policies, scholars have generally relied upon cohort studies (e.g., the Rochester Youth Development study as described in Thornberry et al. (2018); this is also true for research specifically on parental incarceration. It is unknown whether results generalize to other birth cohorts, subgroups, or the full population.

This study employs data from three widely used datasets. The Fragile Families and Child Wellbeing Study (FFCWS) is commonly used because it oversamples populations that are most affected by incarceration, including Black families, Hispanic families, and children born to unmarried parents. However, the FFCWS is not nationally representative^2^ and, although it provides important insight into the incarceration experiences of certain disadvantaged populations, its generalizability is questionable. The Panel Study of Income Dynamics (PSID) and its Child Development Supplement (CDS) have been used to analyze the effects of incarceration on offenders’ life outcomes (Daza et al., 2020), as well as descriptively comment on the circumstances of their children (Johnson, 2009). Nonetheless, as a nationally-representative survey, the PSID has a small sample of persons affected by incarceration and thus does not have much statistical power when the sample is split into subgroups for in-depth analyses (e.g., subgroups by the timing of incarceration). We draw on data from these three large-scale longitudinal samples, which together provide a uniquely comprehensive picture of parental incarceration when compared to past studies.

Our analyses treat parental incarceration as an event that links two life courses and examine the influence of incarceration timing relative to both the child’s and parent’s life courses on children’s educational outcomes. We analyze outcomes at two different stages of children’s developmental growth, their academic abilities during childhood and their long-term educational attainment as measured through years of completed schooling by age 25. We develop counterfactual models with inverse probability treatment weights that address time-varying confounding in the effect estimation of parental incarceration. With these models, we test the effects of parental incarceration timing on children’s education with a particular interest in understanding effect heterogeneity by race/ethnicity.

Our results show evidence across multiple data sets that incarceration before parenthood can lead to poorer educational performance and attainment for children. This finding supports our hypothesis of entwined life events, which emphasizes the possibility that events that shape a person’s life trajectory may or may not happen during the person’s own lifetime. Furthermore, we show that parental incarceration has greater, and often more significant, effects on White children than on Black and Hispanic children on educational attainment and cognitive ability measures. Consistent with prior research findings (e.g., Haskins, 2014), the weaker effects among minority children are associated with greater effect variation. The effect of incarceration depends on the life stage at which the event happens, i.e., before vs. after childbirth, and sometimes on the timing of the event within each life stage. Parental incarceration has a more significant effect on Black children’s educational attainment than on their childhood academic abilities, indicating that unobserved behavioral and institutional factors may play a bigger role in explaining racial gaps in education than cognitive factors. This work also addresses a call to more rigorously assess the effects of parental incarceration on child academic skills. Our results suggest the need for future research and public policies on child welfare and families to consider the past history of family disadvantage that intertwines with present childhood adversity.

### 1.1. A new entwined life events perspective

Within studies of crime and punishment, the life course approach emphasizes the occurrence of criminal behaviors and its effects on offenders and their families (Laub & Sampson, 1993; Sampson & Laub, 1992, 1997, 2003). This approach views life events in the context of life stages that are embedded in social institutions (Elder, 1985). During one’s life course, people experience both long-term patterns of events called trajectories, such as employment, and short-term events called transitions, such as marriage or parenthood. Transitions play a significant role in shaping people’s futures because they serve as turning points that modify and redirect subsequent trajectories (Sampson & Laub, 1990). Thus, their timing in the life course is consequential (Blokland & Nieuwbeerta, 2005; Van de Rakt et al., 2010) and presents situational contexts by which people with similar childhoods end up achieving very different long-term life outcomes. For example, the relative timing of transitions such as marriage (Sampson et al., 2006; Warr, 1998), beginning a job (including even marginal employment) (Uggen, 2000; Wright & Cullen, 2004), and parenthood (Giordano et al., 2011; Pyrooz et al., 2017; Savolainen, 2009; Zoutewelle-Terovan et al., 2014), have been found to explain the differential persistence and desistence of criminal behavior among people of similar backgrounds.

Incarceration has been studied as a major transition that causes permanent effects on individuals’ trajectories (Pettit & Western, 2004; Wakefield & Uggen, 2010), including negative consequences for labor market success and overall wage rates (Huebner, 2005; Pager, 2008; Western et al., 2001), the likelihood of marriage (Huebner, 2005), health (Massoglia & Pridemore, 2015; Massoglia & Remster, 2019) and even recidivism (Gendreau et al., 2000; Spohn & Holleran, 2002). Though not an initial focus of scholars, the collateral effects of incarceration on inmates’ families have been given increasing attention as recent research has come to realize that one’s punishment does not occur in isolation (Braman, 2007; Comfort, 2009; Travis, Western, & Redburn, 2014). The life course literature now treats incarceration of a parent as a major transition in children’s trajectories (Mears & Siennick, 2016). These studies have overwhelmingly linked parental incarceration to negative effects for children, such as antisocial behaviors and delinquency during both child- and adulthood (Murray & Farrington, 2005; Murray & Farrington, 2008; Murray et al., 2012; Swisher & Shaw-Smith, 2015), mental health problems (Mears and Siennick, 2016), and lowered educational outcomes (Haskins, 2014; Turney & Haskins, 2014). Independent of its effects on the incarcerated themselves, parental incarceration is a turning point that shapes children’s lives and needs to be analyzed from both intra- and intergenerational perspectives.

The life course approach clearly places great importance on the timing of events. When examining parental incarceration, studies typically scrutinize the occurrence of this transition in the context of a child’s developmental stage (for example, childhood or adolescence) and numerous other demographic and situational variables, such as family socioeconomic background (Ryabov, 2020), neighborhood disadvantage (Finkeldey & Dennison, 2020a), and even whether children are aware of the incarceration (Woo & Kowalski, 2020). We propose the concept of entwined life events to explain the long-term implications of important life events, such as incarceration, that play out over the course of multiple generations and lives. Such an analytic approach is logical because parental incarceration is not consistently associated with a particular stage of a child’s development, but rather may occur temporally at any point prior or during it. For this reason, parental incarceration is not an age-graded event (Young et al., 2020). Yet, parental incarceration timing, both with respect to developmental stage in which effects are analyzed and relative occurrence to the catalyst’s life course, dictates the social contexts with which the event interacts and the possible mechanisms by which it can be consequential. In summary, an entwined life events perspective is interested in timing for four reasons: First, these events are stage-graded and their effects on actors differ based on the developmental stages in which their effects are evaluated, regardless of when the event actually occurred. Second, these effects are expected to be cumulative, meaning that an early occurrence during the life course is expected to lead to magnified effects as children grow older (Poehlmann-Tynan & Turney, 2021). Third, the relative timing of the event’s occurrence to a child’s life course makes relevant the specific mechanism through which the event impacts the latter. While the present study applies the entwined life events concept to an adverse childhood experience caused by a parent, it is also applicable to positive experiences and events caused by non-parent catalysts, such as a new association with a socioeconomically-advantaged step-parent or close friend. More generally, this perspective offers a means of extending the life course framework to settings where relative timing in multiple lives may be relevant, and the focal event may or may not intersect the focal life course. Fourth, the entwined life event perspective’s focus on timing may also illuminate heterogeneous dynamics that are not evident from other life course approaches. In this work, we look at differences in effects by race as an illustration of this.

### 1.2. The relationship between parental incarceration and children’s educational outcomes

This paper employs the aforementioned entwined life events perspective to examine whether important transitions in another’s life are consequential to others’ lives regardless of whether they occur prior to or during them. Focusing on the transition of parental incarceration, our analysis investigates the relationship between incarceration and children’s educational outcomes. We engage with two research questions: First, we ask whether parental incarceration timing relative to a child’s life course matters for its influence on their childhood academic growth and highest educational attainment. Unlike past research, we study prenatal parental incarceration in addition to imprisonment that occurs during childhood. Second, we ask whether this relationship varies by children’s racial classification. Across these questions, we hypothesize that consequences of parental incarceration will be dependent upon the event’s relative timing in both generations’ lives, and that there may be substantial effect heterogeneity by race.

Existing literature has yielded mixed evidence about parental incarceration’s effects on children. A minority of the literature has found the event to positively affect children. While this literature does not take the position of advocating for increased incarceration as a criminal sanction, it does find that children with incarcerated parents develop resiliency skills that, when compared to their peers, allow them to navigate difficult situations better (Arditti, 2015; Arditti & Johnson, 2020; Miller, 2007; Poehlmann & Eddy, 2013). Aside from these resiliency studies, the majority of research on parental incarceration finds negative collateral consequences on children across the spectrum of outcomes studied. Within a broader family context, this event can be indicative of other serious problems in parental behavior, including parents’ antisocial behavior, drug use, and violence within the family, which together have an ongoing detrimental influence on children during their formative years that continues as they age (Giordano et al., 2019). Research into parental incarceration’s influence on educational outcomes generally falls into this camp: The event depresses children’s educational outcomes at all stages of their development (for example, reviews such as (Haskins et al., 2018; Turney & Goodsell, 2018) discuss education across the life course), and there is evidence that earlier exposure to incarceration is compounded as children grow and leads to cumulative disadvantages (Miller & Barnes, 2015; Turney, 2022; see DiPrete & Eirich, 2006) for a general explanation of mechanisms of cumulative disadvantage). Though the deleterious effects of parental incarceration on children may be mitigated by the availability of school-based resources such as emotional counseling, nursing services, or more highly educated teachers, even these extra resources cannot prevent them (Finkeldey & Dennison, 2020b). Further, parental incarceration not only affects the children of the incarcerated but “spills over” into the attainments of other students (Hagan & Foster, 2012b; see Finkeldey & Dennison, 2020a) for a general explanation of neighborhood-level incarceration effects).

While parental incarceration is not age-graded or only occurs when a child is at a certain age, it is stage-graded or has unique particular effects which depend upon when during a child’s life course it occurs and/or its effects are being observed. For children 3-to-5 years old, parental incarceration reduces multiple measures of school readiness: early learning skills, self-regulation, social-emotional development, and physical health and motor development (Testa & Jackson, 2021). These factors, in conjunction with observed non-cognitive factors, such as increased aggression, inattentiveness, and hyperactivity, lead to higher likelihoods of placement in special education (Haskins, 2014). During elementary school and middle childhood, parental incarceration is associated with lower cognitive skills (math, reading, and other attentional capacities) (Haskins, 2016; Turney, 2017) and behavioral problems (Wildeman & Turney, 2014). Not surprisingly, children of incarcerated adults at this age are more at risk of both grade retention (Turney & Haskins, 2014) and being either suspended or expelled (Jacobsen, 2016). Once they reach adolescence, children of incarcerated adults have lower grade point averages (Nichols et al., 2016) and are more likely to be truant from school (McCauley, 2020; Nichols et al., 2016). They have an increased likelihood of externalizing behaviors (McCauley, 2020; Ruhland et al., 2020), including damaging property, fighting and stealing, and more antisocial peers (Cochran et al., 2018). These circumstances during their adolescence lead children of incarcerated adults to be at a higher risk of dropping out of high school (Cho, 2011) and therefore also less likely to graduate from college (Hagan and Foster, 2012a; Hagan et al., 2020).

Virtually all of the aforementioned literature focuses on incarceration that occurs during a child’s life course, most commonly concurrently with the child’s developmental period studied. Nonetheless, parents may be incarcerated prior to the birth of their children and, once they are incarcerated, this transition is expected to affect all subsequent trajectories in both their own life courses and those of their future children. For example, Dumont et al. (2014) found that the incarceration of women or their romantic partner in the year before birth decreased their likelihood of beginning prenatal care, and that these women were more likely to report partner abuse and rely on Medicaid and governmental assistance for food; thus, they argued that prenatal parental incarceration was a social determinant of health that deserved study in its own right because of its expected influence on disparities in early childhood development. From a policy-making perspective, it is important to distinguish prenatal from postnatal incarceration because the relative timing of incarceration to a child’s life course is determinative of the mechanisms through which this transition’s effects play out. As one potential difference, compared to children whose parents are incarcerated postnatally, children of prenatally incarcerated parents may benefit from a lack of stigma because their parents’ formerly incarcerated status may be less known to community members years after interactions with the criminal justice system have ceased (for explanations of “labeling” children and its effects, see Besemer et al., 2017; Foster & Hagan, 2007; Phillips & Gates, 2011; Shaw, 2016). On the other hand, children of prenatally incarcerated parents may be comparatively disadvantaged if the incarcerated parent has a history of domestic violence since that parent will not have been removed from the household during childhood and therefore children will be directly exposed to this violent behavior (Wakefield & Wildeman, 2011; Wildeman, 2010).

### 1.3. Disparities in the racial dynamics of sentencing and its effects

The likelihood of being incarcerated, and by consequence the likelihood of having a parent incarcerated, is not equal for all children. There are disparities in prosecutions (Shermer & Johnson, 2010; Spohn & Fornango, 2009) and sentencing (Mustard, 2001; Ulmer, 2012) among defendants with different social characteristics. Controlling for a plethora of factors including personal backgrounds, criminal histories, and criminal charges for which sentenced, scholars have found that sentences are harsher for minorities than Caucasians (Everett & Wojtkiewicz, 2002; Mauer, 2011; Mitchell, 2005), for men than women (Doerner, 2012; Embry & Lyons, 2012; Stacey & Spohn, 2006), and that these disadvantages compound so that Black men are sentenced harshest of all (Steffensmeier et al., 1998). As of 2010, more than 10 million American children had experienced parental incarceration at some point in their lives (Schirmer, Nellis, & Mauer, 2009) and during that year, in particular, more than 2.7 million children or 1 in 28 children had an incarcerated parent Trusts (Trusts, 2010). Though showing Americans’ overall penchant for incarceration, these aggregated figures hide the substantial differences in the likelihood that particular children will experience this phenomenon: at that time, 11.4 percent of Black children had an incarcerated parent as compared to only 3.5 percent of Hispanic children and 1.8 percent of non-Hispanic White children.

Mass incarceration’s stratification by race represents a critical axis of inequality, which leads not only to collateral consequences for individuals, but is also borne collectively in acutely disadvantaged communities (Pettit & Gutierrez, 2018; Shaw, 2016; Wakefield & Wildeman, 2011). Western and Pettit (2010) find the social inequality produced by mass incarceration is “sizable and enduring” because prisoners are often drawn from populations that already have the weakest economic opportunities, thus deepening disadvantage by foreclosing on already limited chances for mobility. This inequality persists over generations because unfavorable relative positions of parents become resources that produce further relative losses for subsequent generations (DiPrete & Eirich, 2006; Sharkey, 2008). However, a growing body of evidence disputes this argument of connecting the impact of parental incarceration to only its raw frequency and instead highlights that having an incarcerated parent in-and-of-itself does not explain how individual children experience this event.

Children’s experience of parental incarceration may vary by race because the lives of different races are embedded in social contexts of varying relative advantages and disadvantages. For example, Maguire-Jack et al. (2020) found that White children are exposed to less adverse childhood experiences (ACE)—such as parental incarceration, parental divorce/separation, and extreme economic hardship—than Black and Hispanic children, with Black children not only being the most likely to experience an ACE, but also to experience the most ACEs. These disparities are evident in Turney (2020) finding that 61 % of Black, 51 % of Hispanic, and 40% of White children have endured at least one ACE. As a result, ACEs are concentrated among already vulnerable populations, such as children of color, and can accumulate throughout childhood. While the accumulation of ACEs leads to relative cumulatively lower total outcomes for minority children, the impact on each individual ACE may be lessened; for this reason, it is not clear whether parental incarceration itself will have a unique effect on children that further depresses their outcomes.

In her study of the relationship between parental incarceration and children’s academic abilities during childhood, Turney (2017) concludes that parental incarceration is more deleterious for children with relatively low risks of exposure to it than for children with higher risks. While Turney (2017) did not specifically address racial heterogeneity in effects, others have found that race appears to be a significant moderator in the relationship between ACEs and various children’s outcomes (Poehlmann-Tynan and Turney, 2021). For example, with respect to parental incarceration, Kitzmiller et al. (2020) found that while youths of color living in neighborhoods with high levels of neighborhood disorder experience no incremental change in generalized anxiety and major depressive disorders when a parent is incarcerated, White youths living in non-disordered neighborhoods have higher levels of these disorders. In their study of the effects of parental divorce—another ACE, Brand et al. (2019) found that this event limits White children’s, but not non-White children’s, educational attainment. In our analysis, we scrutinize both racial heterogeneity in the relationship between parental incarceration and children’s educational outcomes, and how this relationship is influenced by relative timings across multiple life courses.

## 2. Data

To ensure a comprehensive understanding of the effects of parental incarceration on children, we use data from two longitudinal datasets, the Panel Study of Income Dynamics and the Fragile Families and Child Wellbeing Study. Each dataset’s strengths supplement the other’s limitations.

### 2.1. Panel Study of Income Dynamics

The Panel Study of Income Dynamics (PSID) is a longitudinal study of families who were first interviewed in 1968. PSID core respondents consist of a nationally representative sample of approximately 2800 households (SRC sample) and a sample of about 2000 low-income households selected from Standard Metropolitan Statistical Areas (SMSAs) in the North and non-SMSAs in the South (SEO sample). The PSID project conducted annual interviews of core family units (FUs) and new families formed by core FU members from 1968 to 1997 and biennial interviews after 1997. The data have been used to analyze individuals’ academic performance and educational attainment (Sharkey, 2008; Song, 2016; Wodtke et al., 2011) as well as the effect of incarceration on ex-offenders’ later life outcomes (Daza et al., 2020).

Data on the academic performance and achievement of children are collected from both the PSID main individual survey and the Child Development Supplement. We measure the educational attainment of each individual in the child generation at age 25 in the main survey and educational performance of a subsample of children between ages 0 and 18 in a child supplementary module. The CDS was first launched in 1997 to provide a broad array of developmental outcomes for children aged 0 and 12. The original sample includes nearly 3600 children in 1997, who were reinterviewed in 2002 and 2007 if they were still active in the PSID panel and within the age range of 0–18. By 2014, almost all original CDS respondents had reached age 17. For this reason, an entirely new sample was collected to follow youth (aged 0–17 in 2014) living in PSID households (PSID 2010a,b, 2012, 2017, 2019). We summarize data availability for children born in different years in Appendix Table A. Cohorts span the years 1968 to 2013.

The present study follows targeted respondents in a longitudinal and genealogical design. We linked PSID children with their parents using the Family Identification Mapping System (FIMS User Manual 2018). Because only parents who are originally PSID respondents are followed over years, their spouses may not be part of the survey if they did not live in the same household after divorce or the end of cohabitation. For this reason, we may have missing information for some parents who were once incarcerated but did not reside in the same household with their PSID children.

### 2.2. Fragile Families and Child Wellbeing Study

A potential limitation of the PSID data is that the sample contains a very small proportion of children with incarcerated or formerly incarcerated parents and did not include a Hispanic sample until very recent years.^3^ For these reasons we employed data from the Fragile Families and Child Wellbeing Study (FFCWS), which has been widely used in previous studies on parental incarceration (e.g, Haskins et al., 2018; Haskins, 2015). The FFCWS follows a cohort of children born in large U. S. cities between 1998 and 2000. The survey oversamples Black and Hispanic families and follows many children who were born to unmarried parents. For these reasons, the FFCWS offers a unique opportunity to assess parental incarceration effects among a sample of young families who may be more affected by the dynamics of interest. We restrict the FFCWS sample to a subsample of 3116 respondents who have valid scores for Woodcock-Johnson assessments, had valid race/ethnicity data, did not have extreme or outlier values for inverse probability treatment weight measures, and remained eligible for the FFCWS sample according to round four FFCWS sample eligibility criterion.

The FFCWS offers several helpful measures. Key outcomes from this data set include the Passage Comprehension, Letter Word, and Applied Problems subtests of the Woodcock-Johnson Assessment. An extensive sequence of questions facilitates mapping the timing of incarceration spells for both mothers and fathers. Furthermore, survey questions concerning the family’s economic environment, as well as several indicators of relationship disruption, offer a means of understanding some of the pathways that may mediate the negative effects of parental incarceration on the assessed child outcomes.

Our analysis consists of three final samples: (1) the PSID sample includes 3174 Black and 3866 White respondents who were born in PSID households and aged 25 and above; (2) the CDS sample includes 2894 Black children and 2640 White children aged 0–18^4^; (3) the FFCWS sample includes 512 White children, 964 Black children, and 587 Hispanic children. Missing values in control variables are resolved using multiple imputation methods that combine estimates based on five imputations. Model estimates based on complete cases and multiple imputed data show similar results of the treatment effects.

## 3. Measures

### 3.1. Outcome variables

The outcomes of interest in this study consist of three childhood educational achievement measures and one adulthood educational attainment measure. We measure childhood outcomes using the Woodcock-Johnson Psycho-Educational Battery-Revised (WJ-R) Tests of Achievement, a standardized assessment of children’s intellectual skills. The original WJ-R comprises a total of nine subscale scores, but only three scales, i.e., the Letter-Word Identification, the Passage Comprehension, and the Applied Problem tests, were administered in both CDS and FFCWS. The Letter-Word Identification scale assesses reading decoding or the ability to apply knowledge of letter-sound relationships in order to recognize unfamiliar words (Wendling et al., 2007). The Passage Comprehension scale assesses both reading comprehension and cloze ability, which is the ability to understand context and vocabulary in order to identify the correct language or part of speech that belongs in a deleted passage. The Applied Problem scale assesses quantitative reasoning, math achievement, and math knowledge. The childhood educational measures are typically reported in four scores: the raw score, the standardized score, the percentile score, and the W score. We report results from standardized scores in the results section.^5^

We measure individuals’ adulthood educational attainment using their highest years of schooling completed at age 25. The coded values vary from 1 to 17 years, with 1 corresponding to completion of the first grade, 12 corresponding to completion of high school, and 16 corresponding to completion of college. The variable is entered as a continuous variable in the models. For individuals whose educational information is unavailable at age 25, we measure it in the next available survey year. This variable is only available in the PSID data and only requested for individuals who were 16 years or older.

### 3.2. Time-varying exposure variable

The exposure or treatment of interest is parental incarceration, which is assumed to be time-varying. We define parents’ imprisonment status based on a few variables in the PSID. Although the questionnaire would not be answered by an individual who was in jail/prison, the PSID survey still collects some information about these individuals from their family members. Specifically, the sequence number variable includes a category for individuals who are in institutions at the time of the interview. Another variable asks why a person is ineligible for the interview and includes a category for individuals in jail or prison.^6^ In addition, in the employment status variable, individuals who are unemployed were asked to indicate whether they are in prison or jail.^7^ By combining these variables, we are able to cross-validate the imprisonment status of an individual from one wave to the next. Before 1979, only household heads’ and wives’ employment statuses were asked. It is possible that our variable definition may underestimate the rate of parental incarceration for early cohorts. Due to the survey design of the PSID, our measure may also omit short spells of incarceration that lasted less than one year before 1997 and less than two years after 1997.

We rely primarily on parent reports of previous incarceration experience and reports of incarceration status on the survey interview dates to construct incarceration measures in the FFCWS. This includes questions concerning whether, when, and for how long a parent may have been incarcerated. We also draw on information that conveys whether a parent was in prison during an FFCWS follow-up interview. Parental incarceration timing measures are constructed based on reports of duration and year of incarceration spell. While paternal incarceration experience is most common, we include maternal experience as well. Qualitative results remain the same when restricting exclusively to paternal incarceration experience.

### 3.3. Covariates

Time-varying covariates refer to variables that change with time and are potentially different as they are measured at different life stages of parents and offspring. Covariate selection was guided mostly by theory with some additional guidance from the Iterative Propensity Score Logistic Regression Model Search Procedure (Moore, Brand, & Shinkre, 2021). This package implements the (Imbens and Rubin, 2015) iterative search procedure that chooses covariates based on gains to the logit log-likelihood function. The final variables included in our analyses are a child’s age, number of children less than 18, living in the south, total family income, parental employment status, home ownership, family income-to-need ratio, family welfare receipt status, and family structure.

We also include time-invariant covariates for variables that occur before or at the birth of the child generation and do not change with the development course of the child. These variables include race, parent’s education and age at childbirth, subsample ID (SRC vs. SEO sample), and child’s birth weight and gender. Appendix B includes a detailed description of all time-varying and time-invariant covariates included in the analysis.

As an additional sensitivity test, we include measures of parents’ academic assessment scores as potential confounders of the relationship between parental incarceration and children’s achievement. CDS children’s primary caregivers, often the mother, were administered the WJ-R Passage Comprehension test in 1997 as a measure of their reading skills. In 1972, all respondents in the core PSID interview were administered the Lorge-Thorndike Sentence Completion Test as an assessment of verbal skills. These measures have been used as measures of parents’ academic skills in prior research (Duffy & Sastry, 2014).

## 4. Methods

We use marginal structural models (MSM) with Inverse Probability Treatment Weighting (IPTW) to estimate the life-cycle effects of parental incarceration on children’s educational performance and attainment. MSM have been increasingly used in social science research to evaluate causal effects in the presence of time-varying exposure, confounding covariates, and outcomes (e.g., Breen & Ermisch, 2017; Killewald & Bryan, 2016; Sampson et al., 2006; Sharkey, 2008; Wodtke et al., 2011). With observational data, conventional regression and matching methods typically fail in the presence of time-varying confounders affected by prior treatments. The method helps address estimation problems caused by the over-control of time-varying confounders and collider bias in longitudinal settings (Elwert & Winship, 2014; Wodtke et al., 2011). The IPTW estimates of a marginal structural model would remain unbiased under the assumption of sequential ignorability (Robins & Hernan, 2009; Robins et al., 2000).

**Fig. 1.**
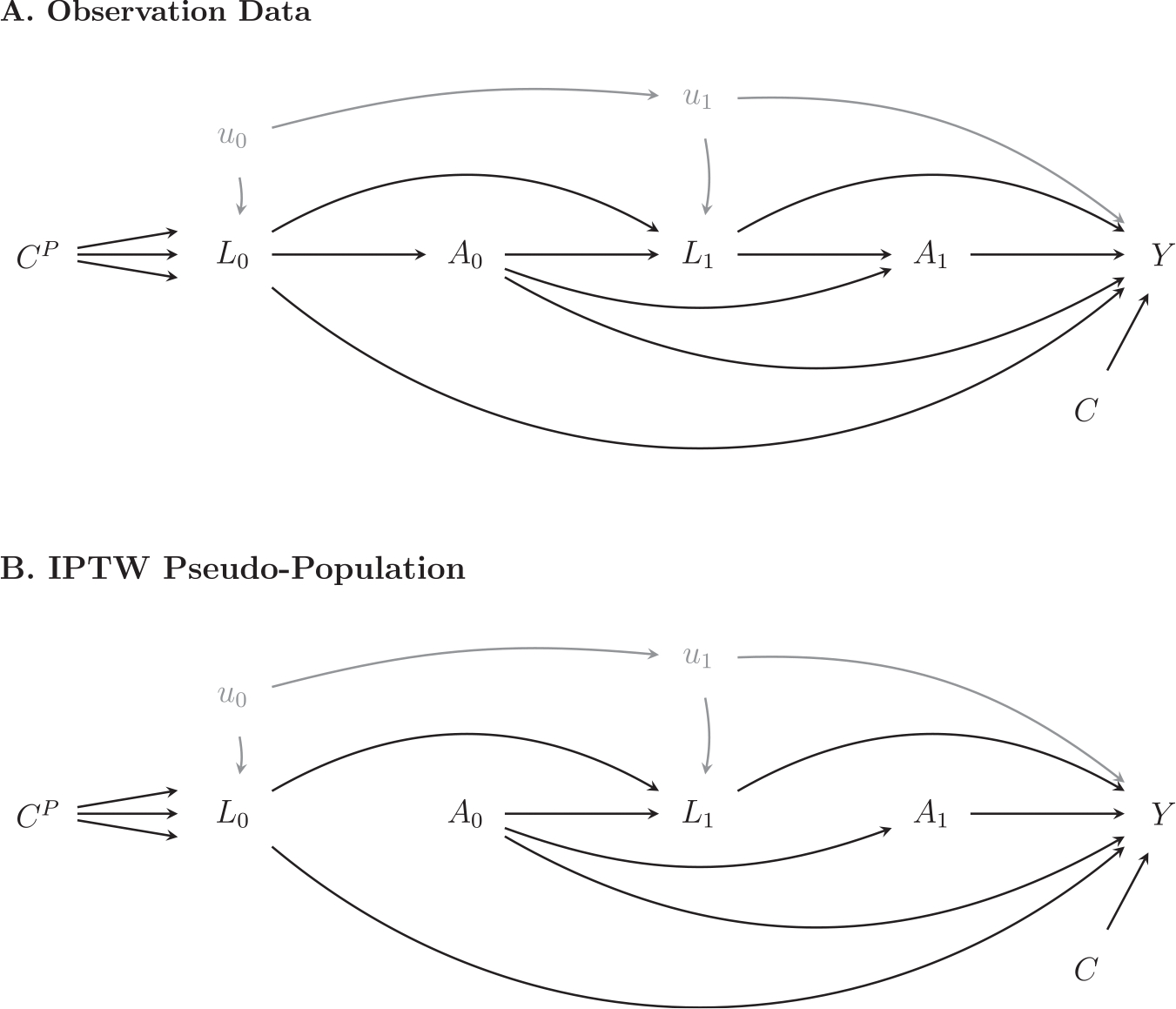
Hypothesized Causal Graphs Before and After the Inverse Probability of Treatment Weighting. *Note: Y* is the academic achievement outcome measured in PSID-CDS or the educational attainment measured in adulthood in the PSID. $A_{0}$ and $A_{1}$ refer to parental incarceration statuses before and after the birth of the child, respectively; We further measure the incarceration timing in FFCWS. ${\bar{L}}_{t}=\{L_{0},L_{1}\}$ is a set of observed time-varying confounders measured for the parent generation before and after the child’s birth. $C^{P}$ and $C_{0}$ refer to time-invariant covariates for the parent generation and for the child generation, respectively. $u_{0}$ and $u_{1}$ refer to time-varying unobserved confounders.

We measure parental incarceration by $\bar{A}=\left\{A_{0},A_{1}\right\}$, where $A_{0}$ refers to pre-childbirth incarceration and $A_{1}$ refers to post-childbirth parental incarceration between age 0 and 18 (or the age when the last CDS measure is observed). Let $Y_{1}$ denote the academic achievement outcome measured in both PSID-CDS and FFCWS and $Y_{2}$ denote the educational attainment measured in adulthood in the PSID. ${\bar{L}}_{t}=\left\{L_{0},L_{1}\right\}$ is a set of observed time-varying confounders measured for the parent generation before and after the child’s birth. $C^{P}$ and $C_{0}$ refer to time-invariant covariates for the parent generation and for the child generation (such as race, gender, and birth cohort), respectively. In our FFCWS analysis, we further allow parental incarceration before and after childbirth to vary by year. The timing effect shows whether the elapsed time between the incarceration event and childbirth moderates the overall incarceration effect. Figure 1 depicts the causal relationships using directed acyclic graphs (DAGs) in the observational data and in a pseudo-population in which treatment variables are no longer confounded by measured covariates, L (see discussions in e.g., Elwert & Winship, 2014; Wodtke et al., 2011). As shown in the figure, after the IPTW weighting, the treatment variables, $A_{0}$ and $A_{1}$, are no longer confounded by the observables L. Panel B in Fig. 1 illustrates the weighting procedure graphically by removing all arrows pointing from L into A. The data structure of the reweighted sample resembles that of a randomized control trial, in which parental incarceration before and after childbirth are both randomized. Compared to propensity score matching or weighting methods, MSM with IPTW is more commonly used for data with time-varying treatment and confounding variables (Hernán et al., 2001; Robins et al., 2000; Wodtke et al., 2011).

Using the notations in the PSID as an example, the IPTW estimates are calculated by fitting the following regression model for childhood outcome, $Y_{1}$,

(1) $$E\left(Y_{1}|A_{0},A_{1}\right)=\alpha_{00}+\beta_{00}A_{0}+A_{1}\left(\beta_{10}+\beta_{11}A_{0}\right)$$

Each object i in the observational data is weighted as follows:

(2) $$w=\prod_{t=1}^{T}w_{t}^{A}=\prod_{t=1}^{T}\frac{P\left(A_{t}=a_{t}|{\overset{‾}{a}}_{t-1},c^{P},c_{0}\right)}{P\left(A_{t}=a_{t}|{\overset{‾}{a}}_{t-1},{\overset{‾}{l}}_{t},c^{P},c_{0}\right)}$$

For the sake of simplicity, the subscript i is omitted in the notation. The denominator of w is the conditional probability that a subject is exposed to his or her actual treatment at each time given prior treatments A and observed confounders L. We estimated a similar model for the outcome $Y_{2}$, i.e., the educational attainment measured in adulthood in the PSID.

## 5. Results

### 5.1. Descriptive statistics

Table 1 presents the parental incarceration rate by race and life stages. In both the main survey and the CDS sample, we observe more parental incarceration among Black than White respondents. Parental incarceration that happened during childhood is more common than before childbirth. The statistics are consistent with previous literature that more Black children have parents who were incarcerated during some time of their childhood than White children: 1.17 % among Black adults aged 25 and above and 0.43 % among White adults in the main survey. We also observe higher incarceration rates in the CDS survey than in the main survey because the CDS sample reflects a more recent trend in parental incarceration than the PSID main survey. About 3.12 % of Black children and 1.54 % of White children experienced parental incarceration during childhood. About 1.14 % of Black children and 0.23 % of White children have parents who were incarcerated at some point before the birth of the CDS children. Note that not all children born into PSID households are included in the CDS sample. Therefore, the percentage of parental incarceration that happened before childbirth does not necessarily refer to the percentage related to first childbirth.

Table 2 provides descriptive statistics of outcome variables and covariates included in our analyses. We summarize the statistics by sample and race. Our dependent variable in the main survey analysis is respondents’ years of schooling at age 25 or above and in the child survey is children’s academic performance test scores (letter-word, passage comprehension, and applied problems). Black people have fewer years of education and lower test scores than White people in both samples. Time-varying covariates show that compared to White respondents, Blacks are more likely to grow up in single-parent families, have parents with work-related disability, have low family income, ever live in poverty, rent their homes, reside in the south, and live in households with more children under 18. Time-invariant covariates show that Black respondents are more likely to come from the low-income SEO sample, have parents with fewer years of education, have younger parents, and have low birth weight than White respondents.

### 5.2. The effect of parental incarceration on academic achievement

The hypothesis of entwined life events argues that significant life events preceding parenthood may affect future child outcomes. We first test this hypothesis by estimating the effect of parental incarceration on academic achievement using PSID–CDS data. Table 3 shows the estimated effects of parental incarceration on academic abilities as measured by the Letter-Word (LW), Passage Comprehension (PC), and Applied Problems (AP) subtests of the Woodcock-Johnson Assessment. Estimates from MSM show significant declines in academic ability scores for White children following parental incarceration. Across all subtests, parental incarceration before birth is associated with academic performance declines of 15 to 20 standardized points for White children. Academic effect estimates for parental incarceration during childhood are generally weaker for White children. Parental incarceration during childhood leads to significant declines in Letter Word Scores for White children by approximately 8.2 points. Effects for the PC and AP subtests were both insignificant. CDS academic ability estimates for Black children are noticeably different. Across all three subtests, there is no evidence of significant test score declines for Black children following parental incarceration before birth. Similarly, CDS estimates offer no evidence of significant declines in academic test scores for Black children following parental incarceration during childhood. This is a sharp departure from the effects observed for White children.^8^

### 5.3. A closer look at the timing effect of parental incarceration

We further test the effect of timing of incarceration during childhood on children’s academic test scores by racial groups. The FFCWS permits estimation of the elapsed time between incarceration spells and the focal child’s date of birth. This allows the estimation of a timing effect that shows whether the timing of parental incarceration spells within the pre-birth and childhood intervals is consequential. We include two sets of variables in Table 4: two dummy variables that measure whether parental incarceration happened before or after childbirth and two continuous variables that measure the number of years before (or after) the childbirth when the parental incarceration first occurred.

Results from Table 4 show that timing effects before birth are generally insignificant across all subgroups for the PC and AP subtests. These results indicate that the magnitude of before-birth parental incarceration effects is generally not sensitive to the timing of parental incarceration within the pre-birth interval. Results for the LW, however, show marginally significant negative effects for both the White and Hispanic subgroups (*p* < 0.1). These negative coefficients convey that pre-birth parental incarceration events that occurred further from the child’s birth are associated with the greatest declines in LW subtest performance.

Timing estimates for parental incarceration during childhood are also mostly insignificant suggesting that the timing of parental incarceration during childhood may not greatly influence the magnitude of academic ability effects. While this is the general case, subgroup-specific estimates reflect more complexity. A significant negative timing effect for White children (*p* < 0.1) on the AP suggests that parental incarceration later in childhood may be more detrimental to their academic ability measures. Conversely, significant positive estimates for Black and Hispanic children on the PC and AP subtests (*p* < 0.05), respectively, indicate that parental incarceration events that happen later in childhood are less detrimental to the academic ability of Black and Hispanic children than events that happen earlier in childhood. Thus, there appears to be a difference in dynamics where parental incarceration events earlier in childhood may be more detrimental for Black and Hispanic children, whereas parental incarceration events that happen later in childhood could have a greater effect on the academic ability of White children. Given that the results do not hold for all three academic achievement measures in our analyses, the effect of incarceration on children’s development may vary in dimensions of skills.

### 5.4. The effect of parental incarceration on educational attainment

In addition to the short-term effects of parental incarceration on children’s academic performance during childhood, we also test whether incarceration has a long-term effect on offspring’s educational attainment in adulthood. Table 5 shows the estimated effects of parental incarceration, both before birth and during childhood, on years of schooling by age 25. We report effects separately for Black and White children with the twofold aims of (1) testing for evidence of entwined life events, and (2) understanding any differences in the dynamics of parental incarceration effects between subgroups. Table 5 shows three sets of results from unadjusted models, regression-adjusted models, and preferred IPT weighted estimates. Specifically, the unadjusted regressions refer to OLS models that only include parental incarceration but no other covariates. The regression-adjusted models further include time-varying and time-invariant covariates shown in Table 2. The IPTW models report estimates from marginal structural models with stabilized probability weighting. For both groups, the unadjusted and regression-adjusted models show more varied parameter estimates with bigger standard errors and similar patterns of statistical significance compared to the IPTW models. Our discussion below focuses on the IPT weighted estimates.

Results in Table 5 for both Black and White respondents offer further evidence of the entwined life events hypothesis. The bottom panel of IPTW stabilized estimates show significant effects of parental incarceration before birth for both Black and White respondents. For the Black group, parental incarceration before birth is associated with a decline of 2/3 years of completed schooling (*p* < 0.05). The corresponding effect for White respondents is noticeably larger, signaling an average decline in completed schooling of 2.9 years (*p* < 0.001). Results also indicate significant negative effects of parental incarceration during childhood for both subgroups. Estimates indicate an average decline in completed schooling of 0.82 years for Black respondents (*p* < 0.1) and 2.3 years for White respondents (*p* < 0.001). In both cases, the standard error estimates suggest no significant difference at the 95% level between the effects of parental incarceration before birth versus during childhood on children’s years of completed schooling. When coupled with the results for academic performance, these estimates suggest that parental incarceration may affect Black children by lowering eventual educational attainment even if there are no corresponding declines in academic ability.

## 6. Discussion

Though social actors lack the agency to initiate or prevent certain events, they are subject to their collateral consequences, which may be either beneficial or detrimental to their life outcomes. Since these actors share close network ties to the event’s catalyst – their parents, siblings, or significant others – their lives are inherently linked to the event apart from and beyond the specific influences of such events on the catalyst’s life course. This motivates our introduction of entwined life events as a framework for analyzing such settings. Examining parental incarceration through an entwined life events framework reveals affect heterogeneity on cognitive outcomes along previously unexplored dimensions. This conveys the salience of contextual considerations concerning timing when characterizing the effects of events that interact with multiple life courses.

Overall, parental incarceration leads to poorer educational outcomes for inmates’ (or ex-inmates’) children in both the short- and long term. During their youth, children of the incarcerated have decreased academic abilities. As these children age, they have an increased likelihood of dropping out and not obtaining a high school or college degree. However, our analysis also shows the variance in how children experience parental incarceration and that this variance is consequential to determine its effect on any particular child’s educational outcomes. Across outcomes, the comparatively advantaged White children in our samples are more impacted by parental incarceration than either Black or Hispanic children. Further, the influence of relative timing varies by children’s racial backgrounds, with significant prenatal effects concentrated among Whites, whereas Black and Hispanic children tend to have poorer educational outcomes when their parents are incarcerated after their births. Using FFCWS data, Wildeman & Turney (2014) found that maternal incarceration is more likely to be significantly associated with behavioral measures for White children relative to Black and Hispanic children. Using the same data, Turney and Wildeman (2015) show that maternal incarceration is most consequential for the well-being of children who were least likely to have maternal incarceration experience. Thus, empirical results here-in contribute to growing evidence that parental incarceration leads to negative effects for different children through different pathways.

Supplementary analyses of the FFCWS data suggest that effect heterogeneity may be driven, in part, by differences in the way specific processes respond to parental incarceration. Differential responses caused by paternal income and family structure illustrate this argument. Evidence from these data shows significant income declines of $35,000 to $60,000 following paternal incarceration for White children while the corresponding estimates for Black and Hispanic children are often insignificant.^9^ Similarly, paternal incarceration is generally uncorrelated with the household structure of Black and Hispanic children while it eventually leads to significantly lower chances of parents remaining together for White children.^10^ These results suggest that parental incarceration can affect salient social processes in different ways across child subgroups, which may motivate affect differences among specific outcomes. Other important processes may also respond heterogeneously to parental incarceration experience in ways that contribute to observed effect heterogeneity on our outcomes of interest. Subsequent work should investigate this possibility more thoroughly.

It is important to reiterate the interpretation of these results relative to the broader incarceration literature. The weaker effects for Black and Hispanic children do not constitute a broader statement against the significance of the prison industrial complex (Davis & Shaylor, 2001) or the carceral state and its disproportional reach into Black, Brown, and poor communities (Hernández et al., 2015; Wildeman, 2009). Similarly, results do not argue against the existence of intergenerational effects of parental incarceration (Western & Pettit, 2010). Instead, this analysis assesses variation in the effects of parental incarceration on children’s cognitive performance according to a specific set of measures. The identified pattern of effect heterogeneity should *not* be interpreted as evidence that parental incarceration is less consequential for Black and Hispanic children. Instead, results convey that declines in cognitive achievement may be more salient for understanding the negative effects of parental incarceration among White children, relative to Black and Hispanic children. From a policy standpoint, this suggests a potential need for different interventions contingent upon the importance of cognitive ability as a pathway that channels parental incarceration effects. Children who show signs of poorer cognitive performance may benefit from interventions that target cognitive growth; other children may have more to gain from interventions that target other dimensions of potential need.

We found no evidence that ex-ante differences between subgroups explain observed effect differentials. Table 2 shows that White children are generally more advantaged than Black and Hispanic children in the FFCWS sample. To better control for this difference in advantage, we re-ran the analysis for Black and Hispanic children from households with above the 50th and 75th percentiles for sample-wide household income. In both cases, the effects of parental incarceration for minority groups were still not comparable to the effects observed for White children. We also consider the possibility that the types of offenses that led to incarceration experience may differ between subgroups. Unfortunately, neither the FFCWS nor the CDS offers data on offense type. At best, we can compare the duration of prison terms as a noisy measure of offense severity. On average, White fathers who were incarcerated before parenthood had slightly longer incarceration terms (109 days) than Black and Hispanic fathers (94 days and 85 days, respectively). These differences are not statistically significant.

By drawing data from multiple large-scale longitudinal samples, our study provides a fuller picture of the impact of parental incarceration when compared to past studies that used only a single dataset. Nonetheless, our analysis may still suffer from several potential limitations. The first limitation of both the FFCWS and PSID is their reliance upon self-reported data. As incarceration is a stigmatized event, there is a possibility that certain respondents do not disclose its occurrence and therefore our results undercount its prevalence. Ideally, self-reported incarceration disclosures would be checked for accuracy against administrative records. However, unlike in certain Scandinavian countries such as Sweden and Denmark, this type of national dataset is not available in the United States. Finally, we examined the sensitivity of our findings with regard to the IPT weight specification. Across multiple weighting specifications, we consistently find evidence of comparable qualitative findings.

Second, despite our preference for differentiating children by the gender of their incarcerated parent, we were not able to do this and maintain statistical power for our results. In all samples, the majority of incarceration events involved paternal, rather than maternal, incarceration. As we sought our results to represent the experiences of the largest number of children captured by our samples, we chose to present analyses for parental incarceration in total. Nonetheless, we ran all models controlling for the incarcerated parent’s gender and obtained results consistent with those presented in this paper since few children had incarcerated mothers. Our robustness checks suggest that the results are driven by paternal incarceration.

Third, we had limited information about the contexts of incarceration because these details were omitted from the datasets. As we argue that the impact on children of parental incarceration is dependent on the circumstances in which the event is embedded, there is a possibility that such omitted variables may explain the heterogeneity in effects that we observed, in particular racial variances. Important omitted variables include the nature of the criminal charge for which one is incarcerated, the length of incarceration (as this determines whether an offender is imprisoned in a local jail, or a state or federal prison) and whether incarcerated parents had contact with children prior to the event. There is also a possibility that some shorter incarceration spells, particularly during the focal child’s childhood, may not be detectable in the data. If undocumented incarceration spells are heterogeneously distributed such that Black and Hispanic parents are more likely to have such experiences, then the weaker effects for these subgroups might be partially attributable to disproportionate contamination among the subgroups specific control (i.e. no parental incarceration experience) groups. The ability to control for these possibilities would have allowed us to confirm that the effects we are attributing to parental incarceration do not have alternative or more nuanced explanations.

Finally, we acknowledge that the relationship between fertility and incarceration is likely more complex than the process modeled above. Our analyses condition upon the child’s conception, birth, and survival through outcome measurement to compare the effects of relative event timing. This analysis does not explore the effects of incarceration on parents’ fertility. While possibilities to conceive a child are highly constrained when men are imprisoned, preliminary evidence from Sykes and Pettit (2009) show that incarceration does not clearly affect lifetime fertility. For male offenders, reduced fertility during incarceration is offset by catch-up fertility upon release. For female partners of the imprisoned, reduced fertility with the incarcerated is offset by increases in the proportion of multiple-partner fertility (Cancian et al., 2016). Future studies may examine the effects of incarceration on fertility decisions, whether the selection process governing fertility of the formerly incarcerated has significant implications for the outcomes of their surviving children, and how these two interlocking processes vary by race.

## 7. Conclusion

The present study offers a new perspective of entwined life events to explain the interconnectedness of life events, outcomes, and trajectories among social actors. Previous life course studies have predominantly focused on the influences of early-life conditions on later-life trajectories within a single generation. Few have examined the ripple effects of one person’s life change on his or her whole social network, within which all members’ social and economic lives are closely intertwined. Such effects depend not only on the catalyst’s own life stage but on the life stages of all actors whose lifetimes may or may not overlap with each other. We develop the term entwined life events to characterize the temporal variance in their occurrence across life courses and further, by the possibility that they may happen prior to the start of one’s life. As our analysis of the entwined life event of parental incarceration demonstrates, the influence of parental incarceration on both children’s academic abilities during childhood and long-term educational attainment is dependent upon when the event transpires relative to their own lives. Children whose parents were prenatally incarcerated are born at a point whereby the cumulative disadvantages associated with imprisonment have already begun to build for their family unit.

It is crucial to remember that individuals facing hardship from entwined life events are being penalized by the collateral consequences of actions that they can neither initiate nor prevent. For this reason, there is an incentive for governments to develop social policies that ensure these events’ effects do not reverberate. Yet, if the role that relative timing plays in shaping these effects is not understood, interventions that are both successful and efficient cannot be crafted. For example, with respect to parental incarceration, scholars have clearly recognized that children of incarcerated parents are an at-risk population and therefore have sought to develop interventions that mitigate the event’s effects on them (discussions of these interventions include Jones et al., 2013; Makariev & Shave, 2010; Miller, 2006; Parke & Clarke-Stewart, 2002). Nonetheless, what is common among these interventions is that they focus solely on children whose parents are incarcerated during their childhood rather than prenatally; the problems encountered by these children are easy for scholars and policymakers to directly observe whereas prenatal incarceration affects future hypothetical children that can only be studied through methods that link their family histories to them posthoc as we have used in this paper. Though interventions are not usually targeted at the future children of the incarcerated, our findings show that for some children, prenatal incarceration is a major transition in children’s lives that potentially has greater negative consequences for their trajectory than if it had occurred during their lifetime. As such, it is imperative that scholars specifically study whether, when, and with whom ex-inmates have children and the mechanisms through which a person’s incarceration history affects the future life course of his or her children from birth in order to develop programs that alleviate the collateral consequences of incarceration.

## Supplementary Material

Appendix

## Figures and Tables

**Table 1:** The percentage of parental incarceration during different life stages.

		Main Survey Age 25 +	CDS Age 0–18	FFCWS Age 0–18

**Black**				
	Before childbirth, %	0.60	1.14	26.62
	During childhood, %	1.18	3.12	35.59
	N	3647	3333	966
**Whites**				
	Before childbirth, %	0.14	0.23	11.99
	During childhood, %	0.41	1.54	10.97
	N	4877	4295	512
**Hispanics**				
	Before childbirth, %	-	-	13.31
	During childhood, %	-	-	21.07
	N	-	-	589

*Source:* Panel Study of Income Dynamics (PSID) Main Survey, 1968–2017; PSID Child Development Supplement (CDS) 1997, 2002, 2007, 2014; Fragile Families and Child Wellbeing Study (FFCWS), 1998–2010. FFCWS estimates are weighted to be nationally representative. *Note:* Parental incarceration during childhood is measured between age 0 and 18 in the main survey and between age 0 and the age when the CDS interview was conducted in the CDS survey. In the unweighted FFCWS sample, incarceration both before *and* after birth occurred for 2–3 % of cases for the White and Hispanic subsamples, and 16 % of cases for the Black subsample.

**Table 2:** Time-Varying and Time-Invariant Sample Characteristics.

	Main Survey				CDS				FFCWS					
	Blacks		Whites		Blacks		Whites		Blacks		Hispanics		Whites	
	Mean	SD	Mean	SD	Mean	SD	Mean	SD	Mean	SD	Mean	SD	Mean	SD

**Outcome variables**														
Y: LW age 0–18	-	-	-	-	97.63	16.81	106.94	17.73	102.28	16.11	97.63	13.11	103.89	13.31
Y: PC age 0–18	-	-	-	-	96.78	15.38	105.24	16.13	90.15	17.28	90.92	12.87	101.11	11.79
Y: AP age 0–18	-	-	-	-	95.96	15.43	107.86	16.64	97.85	14.29	98.22	15.71	107.43	13.17
Y: Years of schooling	12.94	2.01	13.73	2.26					-		-		-	
**Treatment**														
A0: P incarceration, prenatal %	0.60		0.14		1.14		0.23		27.14		13.73		13.04	
A1: P incarceration, age 0–18%	1.18		0.41		3.12		1.54		35.29		20.97		10.96	
**Covariates before or at childbirth**														
L0: P married %	64.00		88.37		42.54		83.86		33.12		60.10		80.43	
L0: P disability %	23.83		13.66		20.37		14.46		0.24		1.15			
L0: H AFDC/TANF receipt %	28.43		7.42		23.19		4.66		33.88		11.29		4.79	
L0: H homeownership %	41.40		67.77		15.84		50.15		34.08		32.28		63.27	
L0: H south residence %	69.56		31.82		64.45		27.19		-		-		-	
L0: H income in 2017 dollars	$28,873	$23,710	$50,880	$36,480	$31,381	$29,301	$63,172	$50,169	$26,320	$26,192	$25,935	$24,072	$60,484	$36,926
L0: H income-to-need ratio	4.00	3.48	7.83	5.90	2.42	2.22	5.03	4.14	1.79	2.07	1.74	1.67	4.49	2.97
L0: H number of children under 18	2.86	2.07	1.89	1.35	2.41	1.48	1.84	1.10	1.26	1.37	1.16	1.17	0.84	1.05
**Covariates during childhood**														
L1: P married %	53.88		76.97		18.66		53.43		34.33		66.78		76.43	
L1: P disability %	49.33		41.83		46.95		38.86		-		-		-	
L1: H AFDC/TANF receipt %	45.79		14.33		36.93		10.45		0.33		0.11		0.05	
L1: H homeownership %	65.86		89.32		25.38		63.59		-		-		-	
L1: H south residence %	72.20		36.76		70.12		34.55		-		-		-	
L1: H income in 2017 dollars	$19,106	$20,813	$39,898	$34,997	$17,650	$20,814	$45,755	$41,761	$29,483	$27,994	$29,574	$25,983	$68,104	$41,820
L1: H income-to-need ratio	1.70	1.89	3.63	3.29	1.06	1.21	2.66	2.60	1.52	1.53	1.44	1.31	3.42	2.15
L1: H number of children under 18	3.64	1.84	2.89	1.31	3.20	1.48	2.75	1.15	1.73	1.61	2.27	1.23	2.11	1.25
**Time-invariant covariates**														
V: C male, %	48.15		50.30		48.21		50.73		59.79		58.89		52.23	
V: H SRC sample, %	12.50		80.78		12.63		86.03		-		-		-	
V: H immigrant sample, %	0.49		4.68		0.96		9.48		-		-		-	
V: H SEO sample, %	87.00		14.54		86.41		4.49		-		-		-	
V: P years of schooling	12.76	1.87	13.41	2.36	13.21	1.75	13.93	2.27	-		-		-	
V: P age of childbirth	23.33	5.36	25.38	5.25	25.15	6.04	27.42	5.54	24.71	6.47	26.65	5.92	28.33	5.93
V: C year of birth	1980.70	6.84	1980.49	6.77	1994.33	6.96	1993.79	6.41	1999.85		1999.94		1999.96	
V: C age at test					9.83	4.02	9.81	3.95	9.21		9.34		9.21	
V: C low birthweight, %					9.33		4.47		-		-		-	
N	3647		4877		3333		4295		1557		815		661	

*Source:* Panel Study of Income Dynamics, 1968–2017; Child Development Supplement 1997, 2002, 2007, 2014; Fragile Families and Child Wellbeing Study (FFCWS), 1998–2017. *Note: Y* = outcome variables, *A_t_* = treatment, *C* = child’s time-invariant covariates, *V* = household-level time-invariant covariates. *H*, *P*, and *C* indicate whether the measure is from household, parent, or focal child, respectively. *LW*, *PC*, and *AP* refer to Letter-Word Identification, the Passage Comprehension, and the Applied Problem tests, respectively. Standard errors are included in parentheses. In the FFCWS, LW was measured in wave 4 during 2003–2006, and PC and AP were measured in wave 5 during 2007–2010.

**Table 3:** MSM Estimated Effects of Parental Incarceration on Academic Achievement, Age 0–18.

	Black			White		
	LW	PC	AP	LW	PC	AP

Parental incarceration before birth	1.993 (2.904)	− 2.709 (2.513)	− 2.318 (2.126)	− 20.174* (8.974)	− 18.860^†^ (10.147)	− 14.990** (5.369)
Parental incarceration during childhood	− 0.465 (1.830)	− 1.472 (1.476)	1.051 (2.343)	− 8.242*** (2.132)	− 4.130 (2.933)	− 5.299 (3.679)
Intercept	97.610*** (0.299)	96.740*** (0.300)	95.833*** (0.275)	107.071*** (0.272)	105.349*** (0.267)	107.934*** (0.257)
Observations	3322	2809	3309	4281	3689	4268

*Source:* Panel Study of Income Dynamics, 1968–2017; Child Development Supplement 1997, 2002, 2007, 2014. *Note:* LW, PC, and AP refer to Letter-Word Identification, the Passage Comprehension, and the Applied Problem tests, respectively. Standard errors are included in parentheses. Coefficients and standard errors are combined estimates from 5 multiple imputation datasets. Other covariates in the model are illustrated in Table 2. The OLS results are presented in Appendix Tables D and E.

† *p* < . 1;

* *p* < . 05;

** *p* < . 01;

*** *p* < . 001 (two-sided tests).

**Table 4:** MSM Estimated Effects of Incarceration Timing (Pre-natal vs. Childhood) on Academic Achievement, Age 0–18.

	White			Black			Hispanic		
	(1a) PC	(1b) AP	(1c) LW	(2a) PC	(2b) AP	(2c) LW	(3a) PC	(3b) AP	(3c) LW

Par. Inc. 0–3 years before birth	− 6.982** (2.466)	− 3.657 (3.291)	− 4.310 (3.861)	− 14.268 (11.716)	− 3.390 (2.601)	− 3.311 (5.060)	0.790 (2.598)	− 2.460 (4.681)	3.077 (5.697)
Par. Inc. 0–9 years after birth	− 10.447** (3.327)	− 8.802* (4.008)	− 5.596* (2.540)	− 4.415* (2.227)	− 4.940^†^ (2.563)	− 6.533 (4.958)	0.419 (4.327)	− 0.483 (4.046)	9.593 (7.702)
Timing. 0–3 years before birth	2.716 (2.526)	0.257 (2.977)	− 8.083^†^ (4.395)	3.200 (4.783)	0.810 (1.465)	7.070 (4.796)	0.641 (1.066)	1.031 (2.436)	− 7.084^†^ (4.274)
Timing: 0–9 years after birth	− 0.540 (0.668)	− 1.131 (0.630)	− 0.821 (1.078)	0.823 * (0.355)	0.479 (0.615)	0.173 (0.481)	0.204 (0.574)	1.494* (0.629)	− 0.877 (1.119)
Intercept	102.900*** (1.249)	108.790*** (1.199)	105.860*** (1.765)	93.541*** (1.673)	100.080*** (1.794)	106.230*** (4.765)	92.235*** (1.735)	99.187*** (2.087)	96.828*** (2.388)
Observations	401	402	259	630	633	457	389	395	254

*Source:* Fragile Families and Child Wellbeing Study 1998–2017. *Note:* Parental incarceration before and after birth refer to dummy variables that show the effect of average change in scores associated with a parental incarceration spell during the specified age-based time interval. Incarceration timing before and after birth refer to continuous variables that show whether the timing within the interval (early vs. later) influences the effect significantly. Estimates show the effect associated with having an incarceration spell one year later in the interval. Standard errors are included in parentheses. Other covariates in the model are illustrated in Table 2. The OLS results are presented in Appendix Tables F, G, and H.

† *p* < . 1;

* *p* < . 05;

** *p* < . 01;

*** *p* < . 001 (two-sided tests).

**Table 5:** MSM Effect Estimates of Parental Incarceration on Years of Schooling at Age 25.

	Black		White	
	Coef	SE	Coef	SE

Parental incarceration before birth	− 0.675*	0.322	− 2.906***	0.640
Parental incarceration during childhood (0–18)	− 0.827^†^	0.451	− 2.306***	0.343
Intercept	12.949***	0.034	13.744***	0.032
Observations	3647		4877	

*Source:Panel* Study of Income Dynamics, 1968–2017. *Note:* Coefficients and standard errors are combined estimates from 5 multiple imputation datasets. The unadjusted model refers to ordinary least square estimates with only parental incarceration variables and without any other covariates. The regression-adjusted and stablized IPTW estimates include time-invariant variables and time-varying variables measured at time 0. Coefficients of these variables are not presented in this table. Full model results are presented in the Online Supplemental Appendix Tables. Other covariates in the model are illustrated in Table 2. The OLS results are presented in Appendix Table I.

† *p* < . 1;

* *p* < . 05;

** *p* < . 01;

*** *p* < . 001 (two-sided tests).

## Footnotes

1 It is worth noting that two studies have included parental incarceration variables that captured prenatal incarceration, though they did not address its effects on outcomes. First, Turney and Lanuza (2017) have a combined variable category for experiencing parental incarceration either prior to birth or after age 17, but this is done in order to separate out incarceration that did not occur during youth and ensure appropriate time ordering. Second, Gaston (2016) parental incarceration variable includes a category for incarceration that occurred either before birth or prior to the child’s first birthday. No reason was provided for this grouping, which we would argue combines events that affect children through different mechanisms.

2 For example, Haskins et al. (2018) discusses how they rarely include nonurban populations.

3 For example, only 1.18% of Black children and 0.41 % of White children in the PSID experienced parental incarceration during childhood (see Table 2).

4 We decided to drop Hispanics and Asians from our PSID sample because there are too few cases for meaningful statistical analyses.

5 The raw score provides a simple count of correct answers in each of the four subtests. Standardized scores are converted from the raw score adjusted for age and scores of members of the child’s age peer group from the WJ-R norming samples. The standardized scores have a mean of 100 and a standard deviation of 15 for each age group. The percentile score provides the percent of children in the same age group who had scores below those of the foci child. Both the standardized scores and percentile scores have been used to compare children of different ages and across different achievement assessments, but they are not designed for evaluating changes in a child’s performance over time because the standardization was implemented for each test independently. The W score is designed for analyzing gains in achievement over time. It is an equal-interval scale that measures both a child’s achievement and the item difficulty on the same scale from a Rasch measurement model. The W score is not dependent upon peer performance. Any 10-point increase in the W score reflects the child’s improvement in ability to perform tasks from 50% success to 75% success.

6 The variable includes separate categories for individuals who are in jail or prison, in the armed forces, in a health care facility, in an educational institution, in some other type of institution (in a religious order or unknown), loss to follow-up, and other reasons.

7 The employment status variable includes separate categories for individuals who are working now, only temporarily laid-off, looking for work, retired, permanently or temporarily disabled, keeping house, student, workfare, or in jail/prison.

8 We have also conducted an analysis by combining parental incarceration before birth and during childhood into a single variable. The results presented in Appendix Tables J and K suggest a significant effect of parental incarceration on children’s academic test scores for all children and a much bigger effect among White children than among Black or Hispanic children.

9 See online Appendix Figure 1 for a graphical representation of this result.

10 See online Appendix Figure 2 for a graphical representation of this result.
