# Supplementary material for: Entwined life events: The effect of parental incarceration timing on children’s academic achievement
Source: Adv Life Course Res. Author manuscript; Available in PMC 2025 Apr 28. (PMC12035912; doi:10.1016/j.alcr.2022.100516)

ONLINE SUPPLEMENT

for
Entwined Life Events: The Effect of Parental Incarceration Timing on Children's Academic Achievement

Appendix A. Data Availability by Birth Cohort in the Child Development Supplement of the Panel Study of Income Dynamics

Birth cohort	Age: Birth			Early childhood				Middle childhood						Adolescence							Educational achievement at age 25
	0	1	2	3	4	5	6	7	8	9	10	11	12	13	14	15	16	17	18	19	
1968	1968	1969	1970	1971	1972	1973	1974	1975	1976	1977	1978	1979	1980	1981	1982	1983	1984	1985	1986	1987	1993
1969	1969	1970	1971	1972	1973	1974	1975	1976	1977	1978	1979	1980	1981	1982	1983	1984	1985	1986	1987	1988	1994
1970	1970	1971	1972	1973	1974	1975	1976	1977	1978	1979	1980	1981	1982	1983	1984	1985	1986	1987	1988	1989	1995
1971	1971	1972	1973	1974	1975	1976	1977	1978	1979	1980	1981	1982	1983	1984	1985	1986	1987	1988	1989	1990	1996
1972	1972	1973	1974	1975	1976	1977	1978	1979	1980	1981	1982	1983	1984	1985	1986	1987	1988	1989	1990	1991	1997
1973	1973	1974	1975	1976	1977	1978	1979	1980	1981	1982	1983	1984	1985	1986	1987	1988	1989	1990	1991	1992	1999
1974	1974	1975	1976	1977	1978	1979	1980	1981	1982	1983	1984	1985	1986	1987	1988	1989	1990	1991	1992	1993	1999
1975	1975	1976	1977	1978	1979	1980	1981	1982	1983	1984	1985	1986	1987	1988	1989	1990	1991	1992	1993	1994	2001
1976	1976	1977	1978	1979	1980	1981	1982	1983	1984	1985	1986	1987	1988	1989	1990	1991	1992	1993	1994	1995	2001
1977	1977	1978	1979	1980	1981	1982	1983	1984	1985	1986	1987	1988	1989	1990	1991	1992	1993	1994	1995	1996	2003
1978	1978	1979	1980	1981	1982	1983	1984	1985	1986	1987	1988	1989	1990	1991	1992	1993	1994	1995	1996	1997	2003
1979	1979	1980	1981	1982	1983	1984	1985	1986	1987	1988	1989	1990	1991	1992	1993	1994	1995	1996	1997	-	2005
1980	1980	1981	1982	1983	1984	1985	1986	1987	1988	1989	1990	1991	1992	1993	1994	1995	1996	1997	-	1999	2005
1981	1981	1982	1983	1984	1985	1986	1987	1988	1989	1990	1991	1992	1993	1994	1995	1996	1997	-	1999	-	2007
1982	1982	1983	1984	1985	1986	1987	1988	1989	1990	1991	1992	1993	1994	1995	1996	1997	-	1999	-	2001	2007
1983	1983	1984	1985	1986	1987	1988	1989	1990	1991	1992	1993	1994	1995	1996	1997	-	1999	-	2001	-	2009
1984	1984	1985	1986	1987	1988	1989	1990	1991	1992	1993	1994	1995	1996	1997	-	1999	-	2001	-	2003	2009
1985	1985	1986	1987	1988	1989	1990	1991	1992	1993	1994	1995	1996	1997CDS	-	1999	-	2001	2002CDS	2003	-	2011
1986	1986	1987	1988	1989	1990	1991	1992	1993	1994	1995	1996	1997CDS	-	1999	-	2001	2002CDS	2003	-	2005	2011
1987	1987	1988	1989	1990	1991	1992	1993	1994	1995	1996	1997CDS	-	1999	-	2001	2002CDS	2003	-	2005	-	2013
1988	1988	1989	1990	1991	1992	1993	1994	1995	1996	1997CDS	-	1999	-	2001	2002CDS	2003	-	2005	-	2007CDS	2013
1989	1989	1990	1991	1992	1993	1994	1995	1996	1997CDS	-	1999	-	2001	2002CDS	2003	-	2005	-	2007CDS	-	2015
1990	1990	1991	1992	1993	1994	1995	1996	1997CDS	-	1999	-	2001	2002CDS	2003	-	2005	-	2007CDS	-	2009	2015
1991	1991	1992	1993	1994	1995	1996	1997CDS	-	1999	-	2001	2002CDS	2003	-	2005	-	2007CDS	-	2009	-	2017
1992	1992	1993	1994	1995	1996	1997CDS	-	1999	-	2001	2002CDS	2003	-	2005	-	2007CDS	-	2009	-	2011	2017
1993	1993	1994	1995	1996	1997CDS	-	1999	-	2001	2002CDS	2003	-	2005	-	2007CDS	-	2009	-	2011	-	-
1994	1994	1995	1996	1997CDS	-	1999	-	2001	2002CDS	2003	-	2005	-	2007CDS	-	2009	-	2011	-	2013	-
1995	1995	1996	1997CDS	-	1999	-	2001	2002CDS	2003	-	2005	-	2007CDS	-	2009	-	2011	-	2013	-	-
1996	1996	1997CDS	-	1999	-	2001	2002CDS	2003	-	2005	-	2007CDS	-	2009	-	2011	-	2013	-	2015	-
1997	1997	-	1999	-	2001	-	2003	-	2005	-	2007	-	2009	-	2011	-	2013	2014CDS	2015	-	-
1998	-	1999	-	2001	-	2003	-	2005	-	2007	-	2009	-	2011	-	2013	2014CDS	2015	-	2017	-
1999	1999	-	2001	-	2003	-	2005	-	2007	-	2009	-	2011	-	2013	2014CDS	2015	-	2017	-	-
2000	-	2001	-	2003	-	2005	-	2007	-	2009	-	2011	-	2013	2014CDS	2015	-	2017	-	-	-
2001	2001	-	2003	-	2005	-	2007	-	2009	-	2011	-	2013	2014CDS	2015	-	2017	-	-	-	-
2002	-	2003	-	2005	-	2007	-	2009	-	2011	-	2013	2014CDS	2015	-	2017	-	-	-	-	-
2003	2003	-	2005	-	2007	-	2009	-	2011	-	2013	2014CDS	2015	-	2017	-	-	-	-	-	-
2004	-	2005	-	2007	-	2009	-	2011	-	2013	2014CDS	2015	-	2017	-	-	-	-	-	-	-
2005	2005	-	2007	-	2009	-	2011	-	2013	2014CDS	2015	-	2017	-	-	-	-	-	-	-	-
2006	-	2007	-	2009	-	2011	-	2013	2014CDS	2015	-	2017	-	-	-	-	-	-	-	-	-
2007	2007	-	2009	-	2011	-	2013	2014CDS	2015	-	2017	-	-	-	-	-	-	-	-	-	-
2008	-	2009	-	2011	-	2013	2014CDS	2015	-	2017	-	-	-	-	-	-	-	-	-	-	-
2009	2009	-	2011	-	2013	2014CDS	2015	-	2017	-	-	-	-	-	-	-	-	-	-	-	-
2010	-	2011	-	2013	2014CDS	2015	-	2017	-	-	-	-	-	-	-	-	-	-	-	-	-
2011	2011	-	2013	2014CDS	2015	-	2017	-	-	-	-	-	-	-	-	-	-	-	-	-	-
2012	-	2013	2014CDS	2015	-	2017	-	-	-	-	-	-	-	-	-	-	-	-	-	-	-
2013	2013	2014CDS	2015	-	2017	-	-	-	-	-	-	-	-	-	-	-	-	-	-	-	-

Note: In 1997, PSID changed every-year interviews to a every other year schedule. Red means CDS-I in 1997, yellow means CDS-II in 2002/2003, blue means CDS-III in 2007/2008, and purple means new CDS in 2014.

Appendix Table B. Stabilized Treatment Weights

	Mean	SD	Range	
			Min	Max
PSID Main Survey				
Blacks	0.998	0.089	0.157	2.955
Whites	0.998	0.049	0.043	2.213
CDS Survey				
Blacks	1.000	0.257	0.009	9.153
Whites	0.982	0.146	0.020	5.369
FFCWS				
Blacks	0.998	0.186	0.449	2.866
Hispanics	0.972	0.304	0.152	2.961
Whites	0.995	0.189	0.318	2.892

Source: Panel Study of Income Dynamics, 1968-2017; Child Development Supplement, 1997, 2002, 2007, 2014; Fragile Families and Child Wellbeing Study, 1998–2017.
Note: These weights are used in Tables 3, 4, and 5.

Appendix C. Measures of Time-Invariant Baseline Covariates and Time-Varying Covariates in PSID, CDS, and FFCWS

Time-varying covariates in our sample include

Age is reported by respondents in each wave of the survey but may not be consistently reported. We first converted individuals' ages into their birth years and created a consistent measure of age using the survey year minus the mode of the birth year measure for each person. We then restricted our sample of the child generation to individuals born after 1968 and thus are younger than 49 in 2017.

Number of Children Less than 18 refers to the number of children from newborns through 17 years of age living in a household, regardless of whether they are actually children of the household head or wife.

Living in the South is a dummy variable coded as 1 if individuals currently live in one of the southern American states (Alabama, Arkansas, Delaware, Florida, Georgia, Kentucky, Louisiana, Maryland, Mississippi, North Carolina, Oklahoma, South Carolina, Tennessee, Texas, Virginia, Washington, D.C., and West Virginia).

Total Family Income refers to the family income reported in the year prior to the survey year. For families that experienced a net income loss, such as a result of business or farm losses, the survey has bottom-coded negative values as $1. Negative values were only bottom coded before 1994 in the PSID. To be consistent across years, we have bottom coded all the negative values. Family income is estimated as the sum of all household members' labor income, income transfers, and social security income. All income variables are converted to 2016 dollars using CPI-U-RS.

Parental Employment Status is measured as whether a parent is employed at the time of a survey. The variable is coded as 1 if both parents are employed and 0 if at least one parent is not employed. Employment status was only collected for PSID household heads and wives before 1979. Given that most parents are either household heads or wives, the missingness is low even before 1979.

Parental Disability is a dummy variable that indicates whether a parent has any physical or nervous condition that limits work ability. This variable is coded from the disability of household head and often refers to a father.

Home Ownership is a dummy variable that indicates whether a family owns or rents their current own. The variable is coded as 1 if a family owns their apartment, mobile home, or house.

Family Income-to-Need Ratio is a common poverty measure, which is calculated as the ratio of total family income to the annual family needs standard from the Census Bureau. The threshold values are adjusted by family size, the number of persons in the family under age 18, and the age of the householder. The needs standard is derived from the poverty threshold published by the Census Bureau (https://www.census.gov/data/tables/time-series/demo/income-poverty/historical-poverty-thresholds.html).

Family Welfare Receipt Status indicates whether a family receive any income from Aid to Dependent children (ADC), Aid to Families with Dependent Children (AFDC), Temporary Assistance

for Needy Families (TANF), or similar state programs. The variable is coded as 1 if a family receives any of these welfare transfers.

Family Structure is a dichotomous variable that indicates whether a child lives in a single-parent or two-parent households at different stages of his or her childhood. A single-parent household refers to families with unmarried or divorced parents.

The time-invariant covariates measured at baseline waves in PSID and FFCWS include

Race is coded 1 for Black respondents and 0 for White respondents in the PSID. Only household head's race is asked in all PSID survey years. We assume that children's race is the same as their household heads. We conduct separate analyses for Black and White children. The PSID survey contains very few Hispanics and Asians in the intergenerational sample. The PSID main sample contains only 44 Asian and 73 Hispanic respondents. The CDS sample contains 71 Asian and 66 Hispanic children. We further include Hispanics in the analysis of the FFCWS sample.

Parent's Education at Childbirth is measured from PSID respondents' highest years of schooling in each wave of the main survey. For most parents, their years of schooling are measured at the year when their child was born. For parents who were incarcerated before the birth of their child, this variable is measured the year when parents were first incarcerated.

Subsamples: The full data include a SRC nationally representative sample, a SEO low-income sample, a Latino immigrant sample collected between 1990 and 1995, and a new immigrant sample added in 1997 and 1999 and followed afterward. To adjust the effect of sampling stratification, we drop the immigrant samples and add a control variable to indicate the sample ID of each family. **?** discusses potential problems of using the SEO sample and the impact on model estimations.

Birth Weight is a dummy variable that is equal to 1 if a child was lower than 2,500 grams at birth and 0 otherwise.

Child's Gender is coded 1 for male and 0 for female.

Parent's Age at Childbirth is measured as the age of the younger parent, often the mother, when the focal child was born. It is calculated as the difference between birth years of parents and children.

Appendix Table D. OLS Estimated Effects of Parental Incarceration on Academic Achievement, White Children Age 0–18

	(1)	(2)	(3)	(4)	(5)	(6)
	LW	LW	PC	PC	AP	AP
Parental incarceration before birth	-9.190	-7.474	-10.750	-11.140†	-3.820	0.694
	(6.296)	(5.803)	(6.576)	(6.167)	(5.915)	(5.438)
Parental incarceration during childhood	-13.620***	-4.448*	-11.070***	-2.503	-11.620***	-1.269
	(2.207)	(2.210)	(2.187)	(2.231)	(2.074)	(2.071)
Male		-1.960***		-1.650**		2.454***
		(0.519)		(0.520)		(0.487)
SRC sample		-		-		-
Immigrant sample		2.191		-1.856		-0.245
		(1.571)		(1.625)		(1.475)
SEO sample		-0.956		-2.629*		-6.929***
		(1.298)		(1.300)		(1.221)
Birth year Flag 1		-		-		-
Birth year Flag 2		-3.231***		-1.820*		-1.185†
		(0.759)		(0.720)		(0.711)
Birth year Flag 3		-0.990		-3.308***		-2.069*
		(0.925)		(0.895)		(0.867)
Birth year Flag 4		-0.452		-3.600**		-0.965
		(1.339)		(1.261)		(1.261)
Birth year Flag 5		4.205**		0.439		1.370
		(1.390)		(1.362)		(1.305)
Birth year Flag 6		-2.550		-		-3.643*
		(1.733)				(1.628)
Mother's age at birth		0.223***		0.107†		0.174**
		(0.059)		(0.059)		(0.055)
Parent's education		1.796***		1.771***		1.883***
		(0.160)		(0.159)		(0.151)
Child age at testing		0.054		-0.840***		-0.172*
		(0.077)		(0.087)		(0.072)
Low birth weight flag		-2.030†		-3.552**		-2.884*
		(1.217)		(1.256)		(1.140)
Parent's married at t0		0.419		0.437		1.280
		(0.925)		(0.939)		(0.870)
Disabled head of household t0		-0.224		0.516		0.506
		(0.812)		(0.805)		(0.762)
Employment status t0		0.440		0.298		1.320
		(1.193)		(1.216)		(1.119)
Income to need ratio t0		0.277		-0.286		0.286

		(0.204)		(0.204)		(0.191)
Number of kids under age 18 t0		-0.976*		-0.678†		0.237
		(0.382)		(0.381)		(0.359)
Homeowner t0		-0.826		-0.404		0.483
		(0.829)		(0.824)		(0.777)
South t0		-0.005		-0.857		-2.491*
		(1.105)		(1.086)		(1.036)
Total family income t0		0.000		0.000*		0.000
		(0.000)		(0.000)		(0.000)
Welfare receipt t0		0.886		0.251		2.857†
		(1.550)		(1.542)		(1.460)
Parent's married at t1		0.174		0.520		0.195
		(0.623)		(0.620)		(0.585)
Disabled head of household t1		-1.594*		-0.856		-0.663
		(0.629)		(0.620)		(0.591)
Employment Status t1		2.969		-0.333		4.349
		(2.941)		(3.293)		(2.756)
Income to need ratio t1		0.713*		0.331		-0.403
		(0.310)		(0.306)		(0.291)
Number of kids under age 18 t1		-0.278		-0.968**		-0.752**
		(0.309)		(0.305)		(0.289)
Homeowner t1		2.270*		2.449**		1.834*
		(0.887)		(0.889)		(0.832)
South t1		2.401*		3.001**		2.750**
		(1.057)		(1.034)		(0.991)
Total family income t1		0.000*		0.000		0.000†
		(0.000)		(0.000)		(0.000)
Welfare receipt t1		-0.592		0.229		-1.690
		(1.193)		(1.167)		(1.121)
Intercept	107.200***	74.030***	105.400***	90.320***	108.100***	70.300***
	(0.272)	(3.923)	(0.267)	(4.162)	(0.256)	(3.677)
Observations	4,281	3,865	3,689	3,309	4,268	3,853

Source: Panel Study of Income Dynamics, 1968-2017; Child Development Supplement 1997, 2002, 2007, 2014.
Note: LW, PC, and AP refer to Letter-Word Identification, the Passage Comprehension, and the Applied Problem tests, respectively. Standard errors are included in parentheses. Coefficients and standard errors are combined estimates from 5 multiple imputation datasets.
$^{\dagger}p < .1$; $^{*}p < .05$; $^{**}p < .01$; $^{***}p < .001$ (two-sided tests).

Appendix Table E. OLS Estimated Effects of Parental Incarceration on Academic Achievement, Black Children Age 0–18

	(1) LW	(2) LW	(3) PC	(4) PC	(5) AP	(6) AP
Parental incarceration before birth	2.869	3.940	0.030	1.126	-0.205	1.124
	(2.775)	(2.548)	(2.660)	(2.408)	(2.552)	(2.462)
Parental incarceration during childhood	-5.074**	-2.179	-2.650	-0.935	0.741	2.745†
	(1.702)	(1.610)	(1.685)	(1.574)	(1.573)	(1.563)
Male		-3.969***		-4.062***		-0.987†
		(0.549)		(0.539)		(0.532)
SRC sample		-		-		-
Immigrant sample		5.265		-0.011		3.330
		(4.503)		(4.245)		(4.350)
SEO sample		0.078		0.387		-0.123
		(0.817)		(0.797)		(0.791)
Birth year Flag 1		-		-		-
Birth year Flag 2		-3.487***		-3.005***		-2.702***
		(0.787)		(0.727)		(0.762)
Birth year Flag 3		0.840		-0.835		-1.231
		(0.958)		(0.901)		(0.929)
Birth year Flag 4		-0.232		-4.426***		-2.201†
		(1.269)		(1.164)		(1.232)
Birth year Flag 5		8.298***		2.040		1.337
		(1.328)		(1.277)		(1.286)
Birth year Flag 6		1.079		-		-2.388
		(1.700)				(1.642)
Mother's age at birth		-0.001		0.046		-0.001
		(0.052)		(0.050)		(0.050)
Parent's education		1.151***		0.923***		0.779***
		(0.177)		(0.175)		(0.172)
Child age at testing		-0.655***		-1.341***		-0.401***
		(0.081)		(0.091)		(0.079)
Low birth weight flag		0.826		-0.339		-1.021
		(0.924)		(0.911)		(0.894)
Parent's married at t0		-0.434		-0.361		0.542
		(0.767)		(0.747)		(0.743)
Disabled head of household t0		1.149		0.762		1.136
		(0.753)		(0.733)		(0.729)
Employment status t0		0.240		0.283		-0.281
		(0.743)		(0.732)		(0.721)
Income to need ratio t0		1.571***		0.713†		1.063**

		(0.383)		(0.373)		(0.370)
Number of kids under age 18 t0		-0.522†		-0.507†		-0.017
		(0.303)		(0.298)		(0.293)
Homeowner t0		-0.564		-0.438		-1.365
		(1.054)		(1.018)		(1.022)
South t0		-1.765		-4.753**		-2.264
		(1.624)		(1.597)		(1.569)
Total family income t0		0.000*		0.000		0.000†
		(0.000)		(0.000)		(0.000)
Welfare receipt t0		1.115		-0.178		0.283
		(0.874)		(0.848)		(0.846)
Parent's married at t1		0.886		0.898		-0.091
		(0.896)		(0.878)		(0.866)
Disabled head of household t1		-0.154		-0.427		-0.605
		(0.640)		(0.621)		(0.619)
Employment Status t1		4.366**		4.278**		3.274*
		(1.336)		(1.332)		(1.287)
Income to need ratio t1		0.253		0.854		0.132
		(0.761)		(0.745)		(0.735)
Number of kids under age 18 t1		-0.346		-0.526†		-0.430
		(0.282)		(0.273)		(0.273)
Homeowner t1		1.897*		1.561†		2.525**
		(0.937)		(0.900)		(0.907)
South t1		2.406		5.387***		1.825
		(1.648)		(1.619)		(1.592)
Total family income t1		0.000		0.000		0.000†
		(0.000)		(0.000)		(0.000)
Welfare receipt t1		-0.728		-0.654		1.128
		(0.854)		(0.828)		(0.826)
Intercept	97.760	85.170***	96.860***	97.480***	95.940***	86.770***
	(0.297)	(3.374)	(0.296)	(3.323)	(0.273)	(3.265)
Observations	3,322	3,117	2,809	2,629	3,309	3,104

Source: Panel Study of Income Dynamics, 1968-2017; Child Development Supplement 1997, 2002, 2007, 2014.
Note: LW, PC, and AP refer to Letter-Word Identification, the Passage Comprehension, and the Applied Problem tests, respectively. Standard errors are included in parentheses. Coefficients and standard errors are combined estimates from 5 multiple imputation datasets.
$^{\dagger}p < .1$; $^{*}p < .05$; $^{**}p < .01$; $^{***}p < .001$ (two-sided tests).

Appendix Table F. OLS Estimated Effects of Incarceration Timing (Pre-natal vs. Childhood) on Academic Achievement, White Children Age 0–18

	(1)<br>PC	(2)<br>PC	(3)<br>AP	(4)<br>AP	(5)<br>LW	(6)<br>LW
Par. Inc. 0-3 years before birth	-7.459**	-1.928	-6.722*	5.901	-9.201*	-0.758
	(2.317)	(3.636)	(2.861)	(4.219)	(3.768)	(4.375)
Par. Inc. 0-9 years after birth	-10.950**	-6.253	-7.480†	-3.200	-8.054**	2.491
	(3.542)	(4.120)	(4.435)	(4.677)	(2.804)	(4.174)
Timing. 0-3 years before birth	3.860	2.797	-3.905	-2.690	0.822	-2.754
	(4.140)	(4.277)	(5.465)	(4.059)	(3.212)	(4.502)
Timing: 0-9 years after birth	0.101	0.099	-0.397	-0.090	-0.414	-0.377
	(0.912)	(1.214)	(0.909)	(0.826)	(0.740)	(0.641)
Focal child is male		-3.277†		-1.199		-1.890
		(1.760)		(2.068)		(2.264)
Year of child's birth=1998		-		-		-
Year of child's birth=1999		-2.367		0.815		14.180
		(3.573)		(5.080)		(24.365)
Year of child's birth=2000		-2.265		1.070		18.310
		(3.347)		(4.327)		(24.735)
Constructed - Mother's age (years)		0.309		-1.937		0.983
		(1.436)		(1.632)		(1.818)
Constructed - Mother's age (years^2)		-0.011		0.025		-0.006
		(0.023)		(0.026)		(0.030)
Parent Ed. Round 1: Less HS		-		-		-
Parent Ed. Round 1: HS or Equiv.		2.227		-8.515		2.446
		(3.820)		(5.868)		(5.633)
Parent Ed. Round 1: Some Coll.		5.708†		-1.003		3.753
		(3.421)		(6.001)		(6.073)
Parent Ed. Round 1: Coll. or Grad		6.830		-4.177		-0.516
		(4.257)		(6.880)		(6.735)
Constructed - Household income (with imputed values)		0.000		-0.00016		-0.000233
		(0.000)		(0.000)		(0.000)
Constructed - Poverty ratio - mother's household income/poverty threshold		-1.851		-0.729		-1.446
		(1.173)		(1.423)		(2.052)
Constructed - # of children under 18 in HH		-0.553		0.019		-4.293*
		(1.286)		(1.686)		(1.709)
Constructed - Is mother cohabiting with baby's father - not including marrieds?		1.647		2.716		5.797
		(4.477)		(3.946)		(6.174)

Mother's Ed. Round 1: Less HS		-		-		-
Mother's Ed. Round 1: HS or Equiv.		6.819†		12.500*		13.280†
		(4.120)		(5.945)		(6.930)
Mother's Ed. Round 1: Some Coll.		7.138		7.615		17.640*
		(4.877)		(6.049)		(7.928)
Mother's Ed. Round 1: Coll. or Grad		18.890**		17.720**		10.030
		(5.923)		(6.080)		(9.732)
Constructed - Is father married to child's mother?		5.244		14.160***		7.857
		(4.427)		(3.960)		(6.279)
Constructed - Household income (with imputed values) Round 1		0.000102		0.000184*		0.000366**
		(0.000)		(0.000)		(0.000)
Any Evidence of abuse from either parent's round 1 interview		5.698		-0.166		-
		(7.935)		(11.051)		
Constructed - Household income (with imputed values) Round 4		0.000157†		0.000148†		0.000
		(0.000)		(0.000)		(0.000)
Constructed - Poverty ratio - mother's household income/poverty threshold		-3.113†		-3.318†		2.036
		(1.646)		(1.829)		(2.699)
Constructed - Number of children under 18 in household		-2.922*		-2.708†		-2.217
		(1.160)		(1.437)		(1.625)
Constructed - Mother living with (not married) child's father at year five		2.985		4.746		-3.598
		(3.989)		(4.833)		(3.540)
Mother's Ed. Round 4: Less than HS		-		-		-
Mother's Ed. Round 4: HS or Equiv.		-11.820**		-4.737		-14.100*
		(4.222)		(5.955)		(6.485)
Mother's Ed. Round 4: Some Coll.		-14.520**		-2.204		-20.840**
		(4.554)		(5.874)		(7.087)
Mother's Ed. Round 4: Coll. or Grad		-19.460***		-4.108		-9.609
		(5.494)		(5.565)		(8.090)
Constructed - Is father married to child's mother at year five?		1.288		0.281		1.204
		(2.908)		(3.373)		(3.699)
Constructed - Father Household income (with imputed values) Round 4		0.000†		0.000*		0.000
		(0.000)		(0.000)		(0.000)
Intercept	103.100***	106.000***	109.100***	131.900***	106.200***	62.090†
	(1.046)	(22.536)	(1.090)	(23.708)	(1.553)	(35.830)
Observations	513	396	515	397	321	256

Source: Fragile Families and Child Wellbeing Study 1998-2017.
Note: Parental incarceration before and after birth refer to dummy variables that show the effect of average change in scores associated with a parental incarceration spell during the specified age-based time interval. Incarceration timing before and after birth refer to continuous variables that show whether the timing within the interval (early vs. later) influences the effect significantly. Estimates show the effect associated with having an incarceration spell one year later in the interval. Standard errors are included in parentheses.
$^{\dagger}p < .1$; $^{*}p < .05$; $^{**}p < .01$; $^{***}p < .001$ (two-sided tests).

Appendix Table G. OLS Estimated Effects of Incarceration Timing (Pre-natal vs. Childhood) on Academic Achievement, Black Children Age 0–18

	(1) PC	(2) PC	(3) AP	(4) AP	(5) LW	(6) LW
Par. Inc. 0-3 years before birth	-8.262	-12.980	-2.834	-1.767	-5.506	3.176
	(6.868)	(8.249)	(2.115)	(2.387)	(4.398)	(2.548)
Par. Inc. 0-9 years after birth	-2.681	-5.343*	-3.367†	-5.381*	-7.842†	-1.041
	(1.880)	(2.659)	(1.957)	(2.716)	(4.295)	(2.948)
Timing. 0-3 years before birth	4.419	5.356	1.727	1.228	5.153	1.934
	(4.736)	(4.627)	(2.195)	(1.711)	(5.970)	(4.181)
Timing: 0-9 years after birth	0.387	0.024	0.095	0.385	0.849*	0.616
	(0.294)	(0.608)	(0.420)	(0.591)	(0.381)	(0.588)
Focal child is male		-4.352†		-1.374		-11.450***
		(2.538)		(1.729)		(2.485)
Year of child's birth=1998		-		-		-
Year of child's birth=1999		-1.700		-0.614		3.460
		(6.239)		(6.341)		(5.401)
Year of child's birth=2000		-2.396		1.434		5.874
		(6.018)		(6.112)		(4.947)
Constructed - Mother's age (years)		0.541		-0.174		-3.669†
		(1.650)		(1.273)		(1.902)
Constructed - Mother's age (years^2)		-0.012		-0.003		0.072*
		(0.028)		(0.022)		(0.034)
Parent Ed. Round 1: Less HS		-		-		-
Parent Ed. Round 1: HS or Equiv.		-3.471		-8.766*		-2.959
		(5.476)		(4.143)		(5.093)
Parent Ed. Round 1: Some Coll.		-4.339		-8.810†		-1.620
		(4.818)		(5.174)		(6.126)
Parent Ed. Round 1: Coll. or Grad		7.132		0.610		-0.626
		(10.819)		(6.587)		(5.988)
Constructed - Household income (with imputed values)		0.000112		0.000148		0.0000784
		(0.000)		(0.000)		(0.000)
Constructed - Poverty ratio - mother's household income/poverty threshold		0.422		-1.161		-0.436
		(2.312)		(1.856)		(1.556)
Constructed - # of children under 18 in HH		-0.567		0.464		0.336
		(1.568)		(1.101)		(1.236)
Constructed - Is mother cohabiting with baby's father - not including marrieds?		3.262		4.185†		-1.754
		(2.747)		(2.343)		(2.734)

Mother's Ed. Round 1: Less than HS		-		-		-
Mother's Ed. Round 1: HS or Equiv.		2.645		7.724		7.480
		(5.569)		(4.713)		(5.754)
Mother's Ed. Round 1: Some Coll.		-7.557		4.892		-1.141
		(7.692)		(5.816)		(6.067)
Mother's Ed. Round 1: Coll. or Grad		-16.330		5.695		-4.075
		(14.911)		(8.960)		(8.969)
Constructed - Is father married to child's mother?		-1.886		4.383		-0.166
		(5.520)		(3.068)		(3.356)
Constructed - Household income (with imputed values) Round 1		0.000114		0.000		0.000
		(0.000)		(0.000)		(0.000)
Any Evidence of abuse from either parent's round 1 interview		1.694		6.576		-10.310*
		(5.229)		(5.524)		(4.938)
Constructed - Household income (with imputed values) Round 4		0.00046*		0.00029*		0.0000221
		(0.000)		(0.000)		(0.000)
Constructed - Poverty ratio - mother's household income/poverty threshold		-6.391†		-4.173*		0.667
		(3.394)		(2.098)		(2.173)
Constructed - Number of children under 18 in household		0.597		-0.611		-2.074*
		(0.977)		(0.911)		(0.869)
Constructed - Mother living with (not married) child's father at year five		2.038		-0.906		-5.718*
		(3.167)		(2.499)		(2.582)
Mother's Ed. Round 4: Less than HS		-		-		-
Mother's Ed. Round 4: HS or Equiv.		7.246		3.764		6.006
		(4.725)		(4.305)		(4.931)
Mother's Ed. Round 4: Some Coll.		7.565†		1.567		6.216
		(4.114)		(3.687)		(4.217)
Mother's Ed. Round 4: Coll. or Grad		11.540		-0.397		1.111
		(7.434)		(7.012)		(8.239)
Constructed - Is father married to child's mother at year five?		-8.120		0.763		5.793†
		(6.687)		(2.608)		(3.197)
Constructed - Father's Household income (with imputed values) Round 4		-0.0001		-0.000		-0.000
		(0.000)		(0.000)		(0.000)
Intercept	92.770***	85.650***	99.600***	102.400***	105.900***	144.900***
	(1.454)	(22.279)	(1.499)	(17.673)	(4.145)	(22.976)
Observations	957	625	964	628	682	454

Source: Fragile Families and Child Wellbeing Study 1998-2017.
Note: Parental incarceration before and after birth refer to dummy variables that show the effect of average change in scores associated with a parental incarceration spell during the specified age-based time interval. Incarceration timing before and after birth refer to continuous variables that show whether the timing within the interval (early vs. later) influences the effect significantly. Estimates show the effect associated with having an incarceration spell one year later in the interval. Standard errors are included in parentheses.
$^{\dagger}p < .1$; $^{*}p < .05$; $^{**}p < .01$; $^{***}p < .001$ (two-sided tests).

Appendix Table H. OLS Estimated Effects of Incarceration Timing (Pre-natal vs. Childhood) on Academic Achievement, Hispanic Children Age 0–18

	(1)	(2)	(3)	(4)	(5)	(6)
	PC	PC	AP	AP	LW	LW
Par. Inc. 0-3 years before birth	1.037	1.672	-1.436	-3.100	2.794	3.406
	(2.303)	(2.542)	(3.384)	(3.770)	(4.471)	(3.986)
Par. Inc. 0-9 years after birth	0.504	-1.759	-4.437	-1.012	3.722	4.149
	(3.857)	(3.338)	(4.565)	(4.000)	(4.107)	(4.576)
Timing. 0-3 years before birth	0.215	1.518	2.855	1.960	-3.745	1.470
	(2.812)	(1.609)	(4.078)	(2.612)	(4.099)	(3.702)
Timing: 0-9 years after birth	0.096	1.039†	0.785	1.705**	-0.349	0.543
	(0.591)	(0.576)	(1.172)	(0.651)	(0.742)	(0.778)
Focal child is male		-2.696		-1.168		-2.658
		(1.972)		(2.578)		(3.130)
Year of child's birth=1998		-		-		-
Year of child's birth=1999		2.391		4.010		-18.040*
		(4.776)		(7.501)		(8.936)
Year of child's birth=2000		1.283		0.014		-9.304
		(3.975)		(3.928)		(7.007)
Constructed - Mother's age (years)		-2.093		-1.475		-0.615
		(1.697)		(2.075)		(2.390)
Constructed - Mother's age (years^2)		0.043		0.028		0.017
		(0.031)		(0.038)		(0.043)
Parent Ed. Round 1: Less HS		-		-		-
Parent Ed. Round 1: HS or Equiv.		-4.391		-5.850		6.231†
		(3.674)		(5.149)		(3.735)
Parent Ed. Round 1: Some Coll.		2.681		2.774		8.861†
		(5.698)		(6.523)		(4.909)
Parent Ed. Round 1: Coll. or Grad		5.936		7.586		20.090***
		(6.467)		(9.671)		(5.530)
Constructed - Household income (with imputed values)		-0.00027†		-0.00026		-0.000225
		(0.000)		(0.000)		(0.000)
Constructed - Poverty ratio - mother's household income/poverty threshold		3.458†		3.421		2.694
		(1.880)		(2.513)		(2.373)
Constructed - # of children under 18 in HH		0.595		0.853		-0.647
		(1.017)		(1.274)		(1.317)
Constructed - Is mother cohabiting with baby's father - not including marrieds?		2.482		-1.813		-0.042
		(3.767)		(4.513)		(3.724)

Mother's Ed. Round 1: Less than HS		-		-		-
Mother's Ed. Round 1: HS or Equiv.		-2.119		0.448		-1.000
		(4.162)		(6.466)		(8.474)
Mother's Ed. Round 1: Some Coll.		-15.590**		-12.240		-3.177
		(5.654)		(7.519)		(8.662)
Mother's Ed. Round 1: Coll. or Grad		-9.859		-31.130**		-2.866
		(6.316)		(10.658)		(13.734)
Constructed - Is father married to child's mother?		1.780		-1.896		0.196
		(4.660)		(5.645)		(4.206)
Constructed - Household income (with imputed values) Round 1		0.000		0.000106		0.000228
		(0.000)		(0.000)		(0.000)
Any Evidence of abuse from either parent's round 1 interview		-7.782†		8.464		-23.300***
		(4.363)		(5.691)		(6.546)
Constructed - Household income (with imputed values) Round 4		0.000159		0.000137		0.000108
		(0.000)		(0.000)		(0.000)
Constructed - Poverty ratio - mother's household income/poverty threshold		-2.204		-1.568		-0.531
		(1.588)		(1.988)		(1.960)
Constructed - Number of children under 18 in household		-0.762		1.080		-0.639
		(0.950)		(1.270)		(1.171)
Constructed - Mother living with (not married) child's father at year five		-0.403		-1.645		6.387†
		(3.367)		(3.730)		(3.767)
Mother's Ed. Round 4: Less than HS		-		-		-
Mother's Ed. Round 4: HS or Equiv.		4.906		2.590		6.108
		(3.609)		(5.389)		(8.156)
Mother's Ed. Round 4: Some Coll.		14.350		9.503†		7.057
		(3.939)		(5.657)		(8.085)
Mother's Ed. Round 4: Coll. or Grad		11.130*		20.820*		2.011
		(5.274)		(9.513)		(11.351)
Constructed - Is father married to child's mother at year five?		3.371		7.468†		9.545**
		(3.314)		(4.060)		(3.503)
Constructed - Father's Household income (with imputed values) Round 4		0.000		0.000		0.000
		(0.000)		(0.000)		(0.000)
Intercept	90.810***	108.600***	99.030***	108.400***	96.900***	99.360**
	(1.616)	(22.436)	(1.674)	(27.419)	(1.900)	(31.066)
Observations	577	384	588	390	369	252

Source: Fragile Families and Child Wellbeing Study 1998-2017.
Note: Parental incarceration before and after birth refer to dummy variables that show the effect of average change in scores associated with a parental incarceration spell during the specified age-based time interval. Incarceration timing before and after birth refer to continuous variables that show whether the timing within the interval (early vs. later) influences the effect significantly. Estimates show the effect associated with having an incarceration spell one year later in the interval. Standard errors are included in parentheses.
$^{\dagger}p < .1$; $^{*}p < .05$; $^{**}p < .01$; $^{***}p < .001$ (two-sided tests).

Appendix Table I. OLS Estimated Effects of Parental Incarceration on Years of Schooling at Age 25

	White		Black	
	(1)	(2)	(3)	(4)
Parental incarceration before birth	-2.745**	-0.907	-0.729	-0.423
	(0.854)	(0.719)	(0.445)	(0.424)
Parental incarceration during childhood	-1.895***	-0.032	-0.233	0.205
	(0.506)	(0.530)	(0.319)	(0.333)
Male		-0.521***		-0.886***
		(0.060)		(0.076)
SEO Sample		-1.247***		-0.150
		(0.093)		(0.113)
Birth year		-0.015†		0.001
		(0.008)		(0.009)
Mother's age at birth		0.037***		0.027***
		(0.007)		(0.008)
Parent's education		0.391***		0.183***
		(0.018)		(0.023)
Parent's married at t0		0.129		-0.120
		(0.145)		(0.129)
Disabled head of household t0		-0.210*		0.023
		(0.098)		(0.100)
Income to need ratio t0		-0.018		-0.010
		(0.012)		(0.024)
Number of kids under age 18 t0		0.007		-0.027
		(0.035)		(0.029)
Homeowner t0		0.111		0.042
		(0.075)		(0.090)
South t0		-0.188		0.201
		(0.120)		(0.224)
Total family income t0		-0.00000066		0.00000354
		(0.000)		(0.000)
Welfare receipt t0		-0.233		0.125
		(0.156)		(0.112)
Parent's married at t1		0.244**		0.462***
		(0.094)		(0.122)
Disabled head of household t1		-0.190**		-0.178*
		(0.067)		(0.084)
Income to need ratio t1		0.003		-0.013
		(0.022)		(0.051)
Number of kids under age 18 t1		-0.076*		-0.029
		(0.031)		(0.029)
Homeowner t1		0.132		0.175†

		(0.120)		(0.097)
South t1		-0.036		-0.159
		(0.115)		(0.230)
Total family income t1		0.00000506*		0.0000104*
		(0.000)		(0.000)
Welfare receipt t1		-0.306**		-0.320**
		(0.116)		(0.104)
Constant	13.740***	37.570*	12.950***	7.802
	(0.032)	(15.631)	(0.034)	(17.830)
Observations	4,877	3,957	3,647	2,413

Source: Panel Study of Income Dynamics, 1968-2017.
Note: Coefficients and standard errors are combined estimates from 5 multiple imputation datasets.
$^{\dagger}p < .1$; $^{*}p < .05$; $^{**}p < .01$; $^{***}p < .001$ (two-sided tests).

Appendix Table J. MSM Estimated Effects of Parental Incarceration Before Focal Child's Birth or During Focal Child's Childhood on Academic Achievement, Age 0-18

	Pooled			Black			White		
	LW	PC	AP	LW	PC	AP	LW	PC	AP
Parental incarceration at any time	-6.451***	-6.814**	-5.714**	0.198	-1.829	0.316	-13.276**	-10.677†	-9.416**
	(1.921)	(2.348)	(1.757)	(1.573)	(1.316)	(1.986)	(4.568)	(5.597)	(3.562)
Intercept	102.933***	101.631***	102.648***	97.605***	96.739***	95.835***	107.072***	105.350***	107.936***
	(0.209)	(0.207)	(0.201)	(0.299)	(0.300)	(0.275)	(0.272)	(0.267)	(0.257)
Observations	7603	6498	7577	3322	2809	3309	4281	3689	4268

Source: Panel Study of Income Dynamics, 1968–2017; Child Development Supplement 1997, 2002, 2007, 2014.
Note: LW, PC, and AP refer to Letter-Word Identification, the Passage Comprehension, and the Applied Problem tests, respectively. Standard errors are included in parentheses. Coefficients and standard errors are combined estimates from 5 multiple imputation datasets.
$^{\dagger}p < .1$; $^{*}p < .05$; $^{**}p < .01$; $^{***}p < .001$ (two-sided tests).

Appendix Table K. MSM Estimated Effects of Parental Incarceration Before Focal Child's Birth or During Focal Child's Childhood on Academic Achievement on Academic Achievement

	Pooled			White			Black			Hispanic		
	LW	PC	AP	LW	PC	AP	LW	PC	AP	LW	PC	AP
Parental incarceration at any time	-2.097	-7.044**	-5.702**	-5.834*	-9.210***	-7.255**	-5.096	-7.617	-3.965†	5.969	0.890	0.808
	(2.156)	(2.256)	(1.770)	(2.748)	(2.088)	(2.580)	(4.970)	(4.823)	(2.178)	(4.529)	(2.672)	(3.344
Intercept	103.487***	98.069***	104.107***	105.852***	102.906***	108.796***	106.209***	93.674***	100.038***	96.777***	92.217***	99.141***
	(1.479)	(1.073)	(1.178)	(1.755)	(1.244)	(1.195)	(4.806)	(1.685)	(1.815)	(2.386)	(1.735)	(2.087
Observations	1008	1481	1491	259	401	402	457	630	633	254	389	395

Source: Fragile Families and Child Wellbeing Study 1998–2017.
Note: LW, PC, and AP refer to Letter-Word Identification, the Passage Comprehension, and the Applied Problem tests, respectively. Standard errors are included in parentheses. Coefficients and standard errors are combined estimates from 5 multiple imputation datasets.
$^{\dagger}p < .1$; $^{*}p < .05$; $^{**}p < .01$; $^{***}p < .001$ (two-sided tests).

Appendix Figure 1: Average Decline in Paternal Income Associated with Paternal Incarceration Before Parenthood Across Survey Waves by Focal Child's Race/Ethnicity

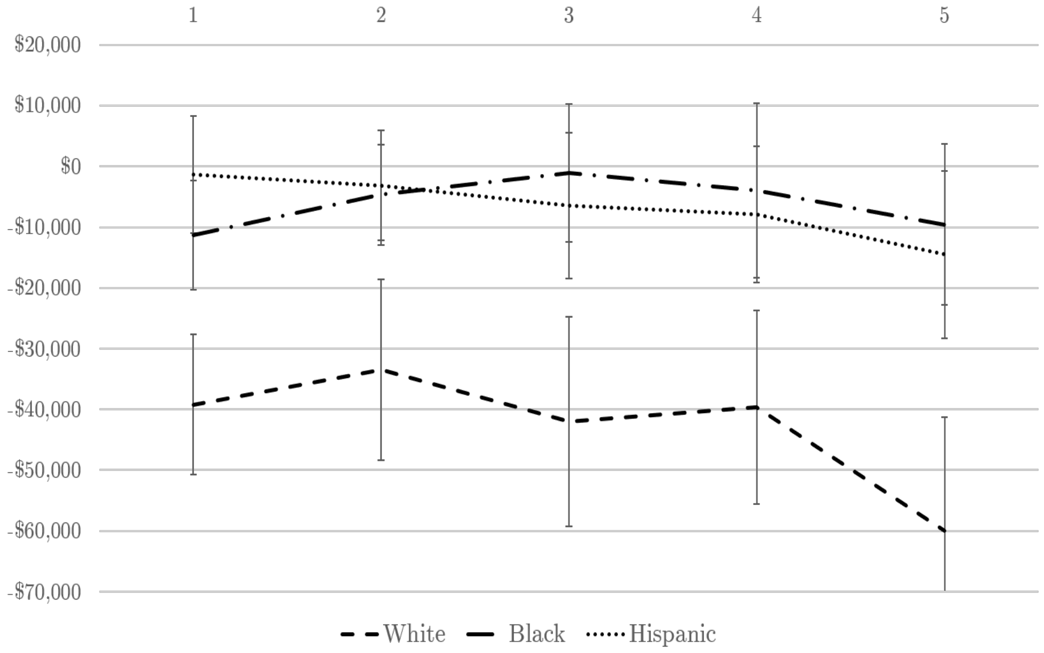

Appendix Figure 2: Average Association Between Parental Incarceration Before Focal Child's Birth and Mother/Father Remaining Involved by Focal Child's Race/Ethnicity

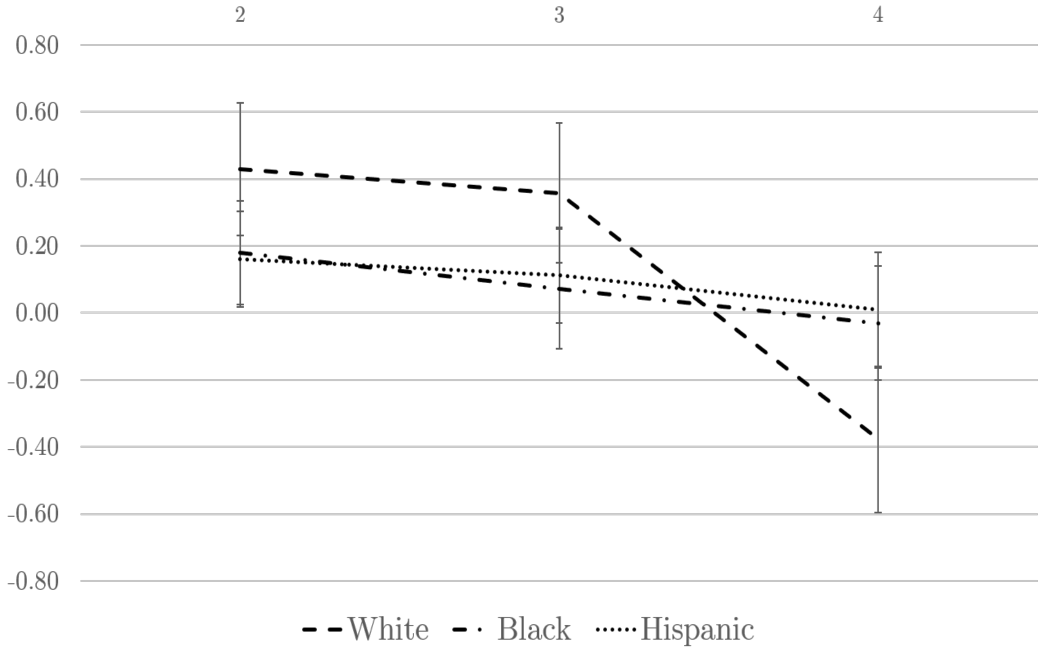

Supplement: Appendix [file NIHMS2056810-supplement-Appendix.pdf]
